# Adsorption Properties and Composition of Binary Kolliphor Mixtures at the Water–Air Interface at Different Temperatures

**DOI:** 10.3390/molecules27030877

**Published:** 2022-01-27

**Authors:** Magdalena Szaniawska, Katarzyna Szymczyk, Anna Zdziennicka, Bronisław Jańczuk

**Affiliations:** Department of Interfacial Phenomena, Institute of Chemical Sciences, Faculty of Chemistry, Maria Curie-Skłodowska University in Lublin, Maria Curie-Skłodowska Sq. 3, 20-031 Lublin, Poland; magdalena.szaniawska@poczta.umcs.lublin.pl (M.S.); katarzyna.szymczyk@mail.umcs.pl (K.S.); anna.zdziennicka@mail.umcs.pl (A.Z.)

**Keywords:** Kolliphor^®^ ELP, Kolliphor^®^ RH 40, adsorption, thermodynamic parameters of adsorption, monolayer composition

## Abstract

The studies on the adsorption properties and composition of the adsorbed monolayer at the water–air interface of the binary Kolliphor^®^ ELP (ELP) and Kolliphor^®^ RH 40 (RH40) mixtures based on the measurements of the surface tension (*γ_LV_*) of their aqueous solution in the temperature range from 293 to 318 K were carried out. The *γ_LV_* isotherms were described by the exponential function of the second order and the Szyszkowski equation as well as predicted by Fainerman and Miller equation. The obtained *γ_LV_* isotherms were analyzed using the exponential function of the second order, the Szyszkowski, Fainerman and Miller as well as independent adsorption equations. The *γ_LV_* isotherms were also used for determination of the Gibbs surface excess concentration of RH40, ELP and their mixture (*Γ*) at the water–air interface as well as the mixed monolayer composition. Based on *Γ* and the constant *a* in the Szyszkowski equation, the standard thermodynamic functions of adsorption were considered. From the consideration dealing with the *γ_LV_* isotherms obtained by us, it results, among others, that these isotherms for the non-ideal solution of macromolecular surfactants mixture can be predicted using the Fainerman and Miller equation. From this consideration, it also results that a simple method proposed by us, based on the isotherms of RH40 and ELP, allows us to predict the composition of their mixed monolayer in the whole concentration range of RH40 and ELP in the bulk phase.

## 1. Introduction

The wide application of surfactants in many industrial areas, agriculture, pharmacy, medicine and everyday life results from their ability to adsorb at the solid–liquid, solid–air and liquid–air interfaces and to form micelles in the bulk phase of the solutions [1,2,3,4]. Indeed, in practice the mixtures of the surfactants, rather than the individual ones, are used [4,5,6,7]. This is due to the specific properties of the mixed adsorption layers at the different interfaces or the resulting mixed micelles that cannot be predicted on the basis of the single surfactant properties.

The properties of the adsorption layers at the interfaces depend on the kind of the surfactants, orientation of their molecules towards the interface, layers thickness and pacing [4]. In some practical applications, the flexibility of the adsorption layers at the water–air or water–oil interfaces plays an important role. It can be expected that such conditions can be satisfied by the surfactant mixtures, the molecules of which are large and branched. Such surfactants include nonionic Kolliphors (known before as Cremophors) which are represented by Kolliphor^®^ ELP (ELP) and Kolliphor^®^ RH 40 (RH40). These surfactants are applied, among others, in pharmacy [8,9]. Their main constituent is triricinoleate ester of ethoxylated glycerol (Scheme S1 in Supplementary Materials). Besides this constituent the others are polyethylene glycol ricinoleate and the corresponding free glycols [10,11]. Three hydrocarbon chains with one -OH group each constitute the hydrophobic part of the RH40 and ELP molecules. One difference between the tail of RH40 and ELP is that in the hydrophobic ELP tail the double bond exists between the carbon atoms. The hydrophilic character of the molecule head results mainly from the presence of oxyethylene groups. Thus, at the first approximation the properties of the hydrophilic part of RH40 and ELP can be compared to those of Triton’s [4,12,13]. However, the RH40 and ELB molecules have a more complicated structure than Triton’s, and for this reason more cases of these molecule orientations are possible at the water–air interface [10,11]. These orientations can influence on the reduction in the water surface tension by the adsorbed RH40 and/or ELP molecules in the surface region. This may be the reason that the literature does not report the unambiguous opinion dealing with the influence of RH40 and ELP on the water surface tension. It is practically impossible to find description or prediction of the isotherm of the surface tension of the aqueous solution of these surfactants at different temperatures. 

As it is known, in practice the mixtures of surfactants are used, but not single surfactants. Therefore, it is important to predict the surface tension isotherms, composition and packing of the mixed monolayer at the water–air interface as well as the thermodynamic parameters of the adsorption of the surfactants mixture at this interface based on the surface tension isotherms of the aqueous solution of the single surfactants being components of the mixture. This issue was the main objective of our studies. These studies were based on the surface tension measurements of the aqueous solutions of ELP and RH40 as well as their mixtures in the temperature range from 293 to 318 K. The obtained isotherms of surface tension were considered in terms of the concentration of RH40 and ELP in the mixed monolayer at the water–air interface and the composition of this monolayer. Different concepts were applied in this consideration. To explain the adsorption properties of the RH40 and ELP mixtures at the water–air interface, its thermodynamic parameters were determined and analyzed.

## 2. Results and Discussion

### 2.1. Surface Tension

The surface tension of the aqueous solution of RH40 and ELP depends on that of water, RH40 and ELP. According to van Oss et al. [14,15,16,17] the surface tension of liquids and solids can be divided into two components. One, which is present in the surface tension of each liquid or solid, results from the Lifshitz-van der Waals intermolecular interactions ($\gamma^{LW}$) and the other one results from the acid-base intermolecular interactions ($\gamma^{AB}$). The acid-base component depends on the electron-acceptor ($\gamma^{+}$) and electron-donor ($\gamma^{-}$) parameters. However, van Oss et al. [14,15,16,17] suggest that the contribution of the dipole–dipole and induced dipole–dipole interactions to the Lifshitz-van der Waals component of the surface tension is smaller than 2%. This indicates that the $\gamma^{LW}$ component of the solid and liquid surface tension depends only on the dispersion intermolecular interactions.

**Figure 1:**
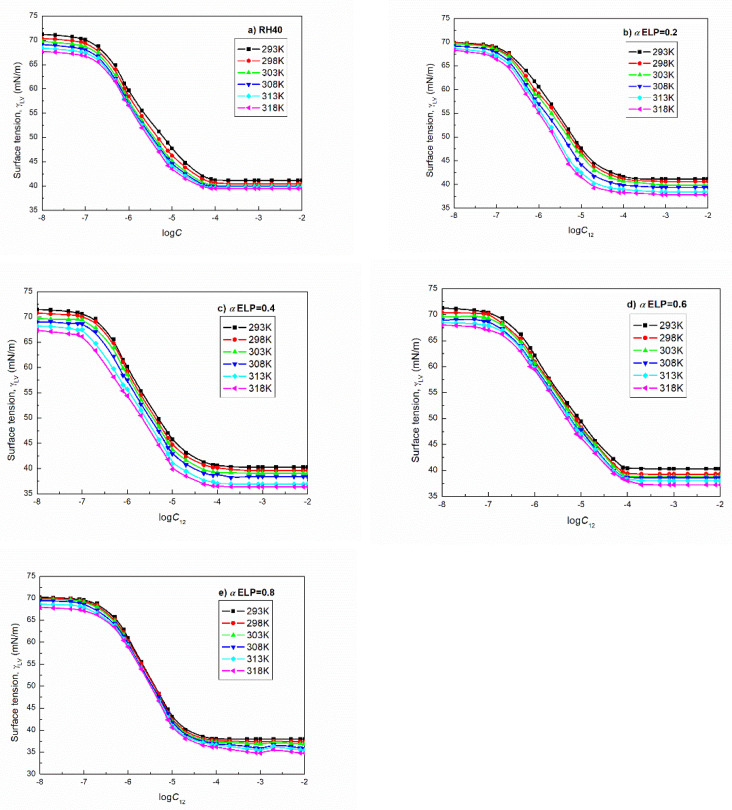
A plot of the surface tension ($\gamma_{LV}$) of the aqueous solutions of RH40 (**a**) as well as RH40 and ELP mixtures at the mole fraction of ELP in the bulk phase, *α*, equal to 0.2 (**b**), 0.4 (**c**), 0.6 (**d**) and 0.8 (**e**) vs. the logarithm of their concentration (logC and $\log C_{12}$) at different temperatures equal to 293, 298, 303, 308, 313 and 318 K, respectively.

Fowkes [18] stated that the dispersion intermolecular interactions at the interface can be deduced on the basis of the interactions of one element with twelve neighbors and the distance between particular elements. In the case of the aqueous solution of RH40 and ELP at the solution–air interface there can be highlighted such elements as follows: water molecule and –CH_3_, –CH_2_–, =CH–, =CO and –OH groups. As the Lifshitz-van der Waals interactions at the first approximation are equal to the dispersion ones, they depend on the type of the groups being in the surfactants molecule, hence their surface tension is related to the orientation of surfactant molecules towards the interface. Taking into account this fact van Oss and Constanzo [15] stated that the surfactant surface tension is different depending on the orientation of its molecules at the surfactant–air interface. Hence, the surface tension of surfactant whose molecules are oriented by tail towards the air phase can be called the “surfactant tail surface tension” ($\gamma_{T}$). In the case of the surfactant molecules orientation by the hydrophilic group towards the air, its surface tension is called the “surfactant head surface tension” ($\gamma_{H}$). If there are no hydrophilic groups and/or a double bond between the carbon atoms in the tail, then the $\gamma_{T}$ results only from the Lifshitz-van der Waals intermolecular interactions. In the case of RH40 the –OH groups present in its tail and in the ELP tail apart from the –OH groups also a double bond occurs between the carbon atoms. For this reason, the hydrophobic properties of tail are reduced. Unfortunately, the literature lacks the data on the surface tension of RH40 and ELP tails and heads. However, it is possible that the surface tension of both surfactants tail is the same and close to the Lifshitz-van der Waals component of fatty acids. Depending on the type of fatty acid the values of $\gamma^{LW}$ are larger than the surface tension of hexadecane and smaller than 30 mN/m. The values of $\gamma_{H}$ of RH40 and ELP can be approximately compared to that of Triton’s, particularly Triton X-165 (TX165), which has 16 oxyethylene groups in one molecule. The $\gamma^{LW}$ value for TX165 is equal to 27.7 mN/m at 293 K [19]. This value is insignificantly higher than that of $\gamma^{LW}$ for the water surface tension (26.85 mN/m) [20], but the electron-acceptor and electron-donor parameters of TX165 acid-base component of its head surface tension differ considerably from those of water surface tension. It seems that the components and parameters of RH40 and ELP surface tension can be close to those of TX165. If so, it can be stated that the adsorption of RH40 and ELP mixture at the water–air interface decreases only the acid-base component of the water surface tension. However, it is possible that in a studied range of mixture concentration the values $\gamma^{LW}$ of the surface tension of surfactant mixtures solution slightly increase as a result of their molecules tail orientation in the mixed monolayer towards the air phase, whereas the reduction in the water surface tension takes place due to the decrease in the acid-base component of its surface tension. In the other words, the number of hydrogen bonds between the water molecules decreases as a function of RH40 and ELP concentration. The greater changes of the number of hydrogen bonds take place in the range of surfactant and their mixtures concentration in which the linear dependence between the surface tension and $\log C/\log C_{12}$ is observed ($C_{12}$ is the mixture concentration) (Figure 1 and Ref. [21]).

**Figure 2:**
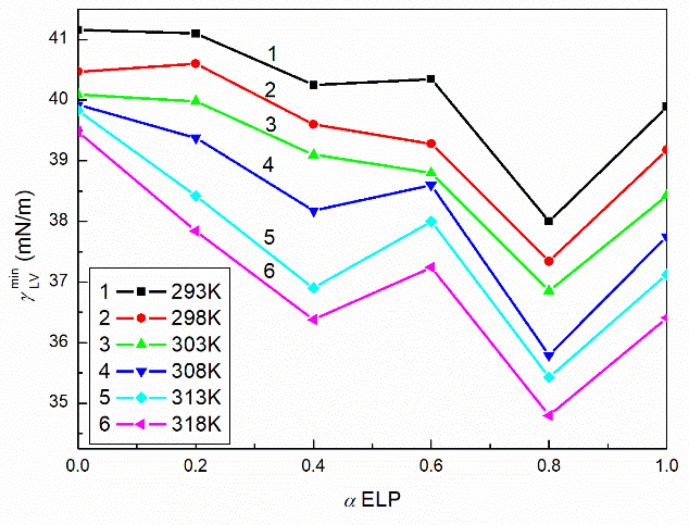
A plot of the minimal surface tension ($\gamma_{LV}^{min}$) of the aqueous solutions of RH40 and ELP mixtures at the temperature equal to 293 (curve 1), 298 (curve 2), 303 (curve 3), 308 (curve 4), 313 (curve 5) and 318K (curve 6) vs. the mole fraction of ELP in the bulk phase, α.

According to the Gibbs isotherm equation [4], the saturated mixed monolayer at the water–air interface is formed in this range of $C_{12}$. The question arises why such a significant decrease in solution surface tension takes place in this concentration range. Indeed, this concentration range is smaller than CMC and it is difficult to take into account the influence of the micelles on the solution surface tension. It is more probable that in the saturated monolayer the surfactant molecules change the orientation and can change the part of tail being in the air phase. This fact causes the changes of the water molecules orientation preventing them from the hydrogen bond formation. On the other hand, the concentration gradient of surfactants at the interface changes with their concentration changes in the bulk phase. As a matter of fact, the destruction of hydrogen bonds is not complete because the minimal surface tension of the aqueous solution of RH40 and ELP mixtures is considerably higher than that resulting from the surfactants tail (Figure 1b–e). The minimal values of the surface tension change as a function of the mixture composition (Figure 2). These changes are not linear and probably result from the synergetic effect of the micelle formation. It is known that only single molecules adsorb at the water–air interface and the surface tension of the surfactant aqueous solution depends largely on the concentration of surfactants in the monomeric form in the bulk phase. This concentration depends on the tendency of the surfactants and their mixture to form micelles. Unfortunately, based only on the surface tension isotherms it is difficult to explain more precisely changes in the minimum surface tension of the aqueous solutions of the RH40 and ELP mixture as a function of its composition. Additional measurements are required. However, we can state that the minimal values of the surface tension of the aqueous solution of RH40 and ELP mixtures do not differ significantly from the minimal values of the aqueous solution of TX165 at a given temperature (39.5 mN/m at 293 K) [22]. However, this value is achieved at a significantly smaller concentration than that of TX165. This indicates that the adsorption activity of RH40 and ELP as well as of their mixture is larger than that of TX165.

The adsorption activity of a surfactant is closely related to the hydration of tail and head of surfactant molecules. Tail hydration is the driving force for the surfactant adsorption and the head hydration is the brake. It was proved that the volume of the surfactant molecule can be approximated by cubes in which a given part of the surfactant is inscribed. Hence, taking into account the length of the bonds and angle between them as well as the distance between molecules, it is possible to establish the contactable area of RH40 and ELP tail as equal ≈967.7 Å^2^. Since the contact plane of the water molecule with a molecule of another substance can be assumed to be equal to 10 Å^2^, theoretically about 97 water molecules can be contacted with the RH40 or ELP tail simultaneously. On the other hand, it is known that one oxygen atom in the oxyethylene group can form a strong hydrogen bond with two molecules of water [23]. Assuming that the other oxygen atoms, except the one in the –OH group, can also form the hydrogen bond with two molecules of water, then the RH40 head can by hydrated by 92 molecules of water and head of ELP by 82 ones. This is in accordance with the earlier studies [24].

If, during the transfer of RH40 and ELP molecules from the bulk phase to the mixed surface layer, the head of their molecules does not exist or is poorly dehydrated, then the driving forces of adsorption are large, and therefore RH40 and ELP are more surface active than Triton’s [25]. It is possible that the dehydration of their head increases with the increasing temperature. On the other hand, the isotherms of the surface tension of the aqueous solutions of RH40 and ELP mixtures with different compositions obtained from the measurements are almost parallel (Figure 1b–e).

**Figure 3:**
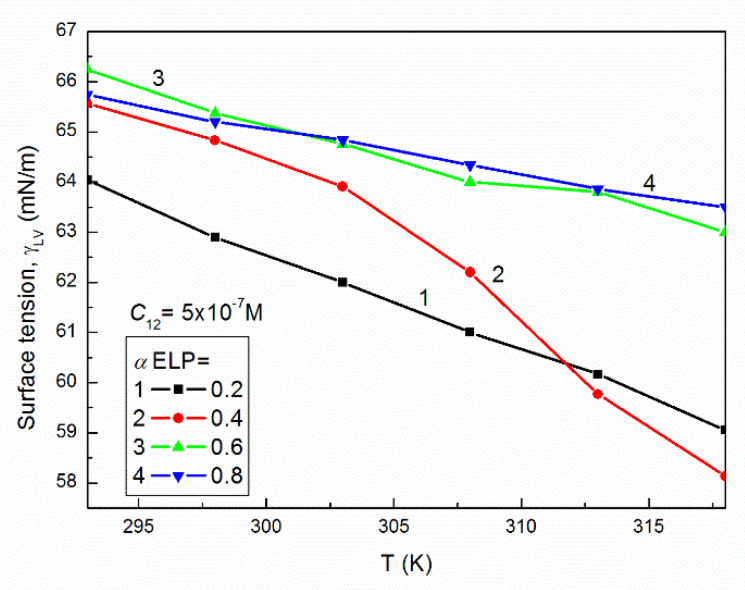
A plot of the surface tension ($\gamma_{LV}$) of the aqueous solutions of RH40 and ELP mixtures at the mole fraction of ELP in the bulk phase equal to 0.2 (curve 1), 0.4 (curve 2), 0.6 (curve 3) and 0.8 (curve 4) at *C*_12_ = 5 × 10^−7^ M vs. the temperature, T.

The surface tension of the aqueous solution of studied mixtures at a given concentration and composition as a function of temperature in most cases decreases almost linearly (Figure 3 as an example). This indicates that in the studied range of the temperature the dehydration of RH40 and ELP is insignificant.

**Figure 4:**
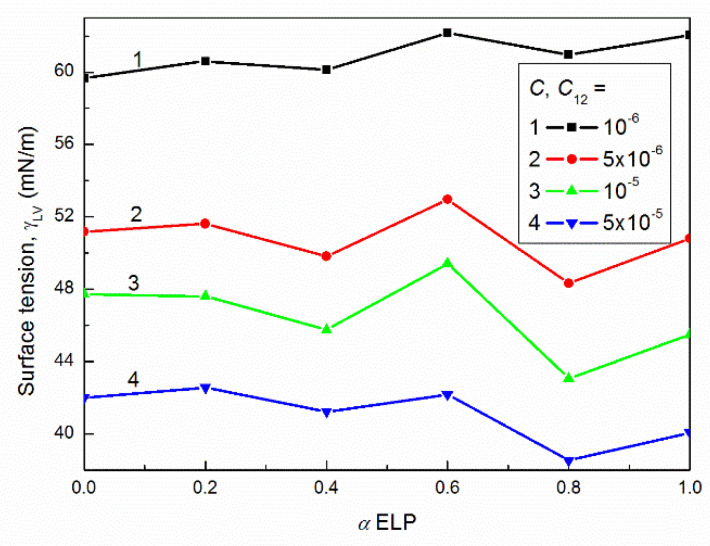
A plot of the surface tension ($\gamma_{LV}$) of the aqueous solutions of RH40 and ELP mixtures at temperature T equal to 293 K and *C* or *C*_12_ equal to 10^−6^ (curve 1), 5 × 10^−6^ (curve 2), 10^−5^ (curve 3) and 5 × 10^−5^ M (curve 4) vs. the mole fraction of ELP in the bulk phase, α.

On the other hand, there is not a linear dependence between the surface tension of the aqueous solution of RH40 and ELP mixture at a given concentration as a function of mixture composition (Figure 4 as an example). The positive and negative deviations of $\gamma_{LV}$ values from the linear dependence were observed. This indicates that some changes in the hydration number of surfactants head can take place and/or the changes in the surfactant molecules packing as a result of their configuration variation. As follows from the literature [26,27] the possibility of forming different configuration by surfactants having large and branched molecules is greater than for the classical surfactants whose molecules are linear and not very long. It is possible that the oxyethylene and/or hydrocarbon chains are coiling. Such phenomenon was observed for other surfactants having many oxyethylene groups in their molecules [28,29].

From the theoretical and practical points of view it is interesting to describe and/or to predict the isotherm of the surface tension of the aqueous solution of the RH40 and ELP mixture on the basis of the isotherm of the individual component of the mixture. It appeared that all isotherms of $\gamma_{LV}$ can be described by the exponential function of the second order (Figures S1–S6 in Supplementary Materials). This function has the form:

(1) $$\gamma_{LV}=y_{0}+A_{1}exp\left(\frac{-C}{t_{1}}\right)+A_{2}exp\left(\frac{-C}{t_{2}}\right),$$

where $\gamma_{LV}$ is the surface tension of the aqueous solution of surfactant, C is the surfactant concentration, $y_{0}$, $A_{1}$, $A_{2}$, $t_{1}$ and $t_{2}$ are the constants.

Unfortunately, no unequivocal dependence of the constants in Equation (1) on the physicochemical properties of surfactants has been reported so far. It was suggested that these constants are related to the components and parameters of the surfactants head and tail [30]. However, it is difficult to predict the constants in Equation (1) for the mixtures of surfactants based on the constants for individual components of the mixtures. In the case of RH40 and ELP mixtures the additional difficulties result from the fact that they can be treated not as binary but rather as multicomponent ones. However, it can be stated that the values of the $y_{0}$ constant are close to the minimal values of $\gamma_{LV}$ at each temperature (Figure S7). As the minimal values of $\gamma_{LV}$ depend on the surface tension of surfactant tail, it can be stated that $y_{0}$ also is related to the tail of surfactant surface tension. Although the $y_{0}$ values are close to the minimum $\gamma_{LV}$ values, the $y_{0}$ values for the RH40 with ELP mixture cannot be predicted based on the $y_{0}$ values for RH40 and ELP. This results from the fact that there is no linear relationship between the $y_{0}$ values and the composition of the mixture (Figure S8). Changes of the remaining constants from Equation (1) are also non-linear as a function of the mixture composition (Figures S9–S12). Hence, it can be concluded that the isotherms of the surface tension of the aqueous solution of RH40 and ELP mixtures can be satisfactorily described but not predicted.

The changes of the surface tension of the aqueous solutions of many surfactants as a function of their concentration can be described by the Szyszkowski equation if the Gibbs surface excess concentration of the saturated monolayer at the water–air interface and the standard Gibbs free energy of adsorption are known. The Szyszkowski equation for the aqueous solution of RH40 and ELP mixtures can be expressed in the form [4]:

(2) $$\gamma_{W}-\gamma_{LV}=\pi =-RT\Gamma^{max}ln\left(\frac{C}{a}+1\right),$$

where $\gamma_{W}$ is the water surface tension, π is the monolayer at the water–air interface pressure, a is the constant, which depends on the standard Gibbs free energy of adsorption ($\Delta G_{ads}^{0})$, $\Gamma^{max}$ is the maximal Gibbs surface excess concentration of surfactant in the saturated monolayer at the water–air interface, *T* is the temperature and R is the gas constant.

The a constant in Equation (2) fulfills the expression [4]:

(3) $$a=\omega \;exp\left(\frac{\Delta G_{ads}^{0}}{RT}\right),$$

where: ω is the number of the water moles in one dm^3^.

To describe the surface tension isotherm Equation (2) can be solved numerically choosing proper values of $\Gamma^{max}$ and a. For calculation of $\gamma_{LV}$ from Equation (2) it was assumed that *C* changes from 0 to CMC. This resulted from the fact that not aggregated surfactant molecules are surface active. It appeared that at the first approximation it was possible to describe all isotherms of surface tension using the Szyszkowski equation (Equation (2)) (Figures S1–S6).

For the ideal mixture of surfactants, it is possible not only to describe but also to predict the isotherm of the aqueous solutions of this mixture at different compositions based on the data for individual surfactants. In the case of the ideal surfactant mixture the changes of the $\Gamma^{max}$, a and CMC as a function of its composition should be linear. In addition, the surface tension of the aqueous solution of ideal surfactants mixture should satisfy the equation which for the binary mixture has the form:

(4) $$\gamma_{LV}=\gamma_{LV}^{1}X_{1}^{S}+\gamma_{LV}^{2}X_{2}^{S},$$

where $\gamma_{LV}^{1}$ and $\gamma_{LV}^{2}$ is the surface tension of the aqueous solution of surfactants 1 and 2 at a given concentration in the bulk phase and $X_{1}^{S}$ and $X_{2}^{S}$ are the mole fractions of surfactants 1 and 2 in the monolayer.

As it was suggested earlier, $X_{1}^{S}$ and $X_{2}^{S}$ can be determined using the film pressure of surfactants 1 and 2 at their given concentration in their individual solutions. Hence, $X_{1}^{S}=\frac{\pi_{1}}{\pi_{1}+\pi_{2}}$ and $X_{2}^{S}=\frac{\pi_{2}}{\pi_{1}+\pi_{2}}$ ($\pi_{1}$ and $\pi_{2}$ are the layer of surfactants 1 and 2 pressure, respectively). The values of $\gamma_{LV}$ calculated from Equation (4) for all studied systems are higher than the measured ones at the same RH40 and ELP concentration (Figures S2–S5). However, the difference between the measured and calculated values of $\gamma_{LV}$ depends on the composition of this mixture. In the case of RH40 and ELP mixture with the mole fraction of ELP equal to 0.6, the calculated values of $\gamma_{LV}$ are almost identical to those measured (Figure S4). For the aqueous solution of RH40 and ELP at a given composition the difference between the calculated from Equation (4) and measured values of $\gamma_{LV}$ practically does not change as a function of temperature (Figures S2–S5).

The difference between the measured and calculated values of $\gamma_{LV}$ indicates that the RH40 and ELP mixture does not behave as ideal one and the deviation from the ideal behaviour depends on the mixture composition. This conclusion is also confirmed by the $\Gamma^{max}$ values used in the Szyszkowski equation (Equation (2)) for the $\gamma_{LV}$ calculations. The $\Gamma^{max}$ values do not change linearly as a function of mixture surfactants composition (Figure S13), and they are in the range from 2.44 × 10^−6^ to 3.12 × 10^−6^ mol/m^2^. These values correspond to the minimal area occupied by one molecule which is in the range from 53.2 to 68 Å^2^. Thus, it can be concluded that there is a synergetic effect in the reduction in water surface tension by the RH40 and ELP mixture which depends on its composition. For this reason, for the RH40 and ELP mixture the surface tension of its aqueous solution cannot be precisely predicted based on the $\Gamma^{max}$, a and CMC for RH40 and ELP. However, this tension can be predicted using the Fainerman and Miller equation. This equation for binary mixtures has the form [31,32]:

(5) $$exp\Pi =exp\Pi_{1}+exp\Pi_{2}-1,$$

where Π=πϖ/RT, $\Pi_{1}=\pi_{1}\varpi_{1}/RT$ and $\Pi_{2}=\pi_{2}\varpi_{2}/RT$ are the dimensionless pressure of the mixed monolayer at the water–air interface and individual surfactants 1 and 2, respectively, and $\varpi_{1}$, $\varpi_{2}$ and ϖ are the areas occupied by one mole of surfactants 1 and 2 and mixture at the water–air interface and π , $\pi_{1}$ and $\pi_{2}$ are the differences between the surface tension of the solvent and solution of the surfactants mixture and components 1 and 2, respectively.

The calculation made using Equation (5) confirmed the above mentioned conclusion that, on the basis of the Fainerman and Miller equation, the isotherm of surface tension of RH40 and ELP mixture can be predicted (Figures S2–S5). However, at the concentration of the surfactant mixtures above their CMC the values calculated from Equation (5) are smaller than those measured. Some differences between the measured and calculated from Equation (5) $\gamma_{LV}$ values are observed for the mixture with the mole fraction of ELP equal to 0.6 at the constant temperatures 313 and 318 K (Figure S4).

### 2.2. Concentration of Surfactants at the Water–Air Interface and Composition of the Mixed Monolayer

In most cases the concentration of surfactants and/or their mixture is determined using the Gibbs adsorption isotherm Equation [4]. As a matter of fact, this equation allows us to determine only the surface excess concentration of surfactants at the interface, but it can be treated as the total dimensional concentration because of the small surfactant concentration in the bulk phase. For the studies on the adsorption properties of surfactants their very low concentration in the aqueous solution is used. Therefore, for such solution it can be assumed that the activity coefficient of surfactants is close to unity and its mole fraction is very close to $\frac{C}{\omega }$ and ω is practically constant in the range of studied surfactant concentrations. In such case the Gibbs isotherm equation for the nonionic surfactants and their mixtures has the form [4]:

(6) $$\Gamma =-\frac{C}{RT}{\left(\frac{\partial \gamma_{LV}}{\partial C}\right)}_{T}=-\frac{1}{2.303RT}{\left(\frac{\partial \gamma_{LV}}{\partial logC}\right)}_{T}.$$

The calculated values of Γ indicate that the adsorption of RH40 and ELP and their mixtures depends on the temperature and in the case of mixtures also on their composition (Figure S14). However, for the RH40 and ELP mixtures in which the mole fraction of ELP is equal to 0.2 and 0.8, respectively, the surface excess concentration at the water–air interface decreases and increases as a function of temperature. This indicates that for these mixtures the synergetic and/or antagonistic effect in the reduction in water surface tension takes place. It is also confirmed by the fact that there is no linear dependence between $\Gamma^{max}$ and composition of the RH40 and ELP mixtures. Thus, the negative and positive deviations from the linear dependence are observed. In many cases the $\Gamma^{max}$ values obtained from the Gibbs isotherm equation using the linear dependence between the surface tension of the solution and the logarithm from the surfactants concentration are similar to the $\Gamma^{max}$ values obtained from the Szyszkowski equation (Figure S13). As mentioned above this equation was solved against the surface tension of the aqueous solution of surfactant numerically.

The calculations of Γ from Equation (6) also indicate that in many cases the values of $\Gamma^{max}$ are obtained for RH40 and ELP as well as their mixtures at the concentration equal to 1 × 10^−6^ mol/dm^3^. It was proved that in the range of the surfactants and their mixture concentrations from 0 to 1 × 10^−6^ mol/dm^3^, the independent adsorption at the water–air interface takes place (Figures S2–S5). This results from the calculation of $\gamma_{LV}$ from the following expression:

(7) $$\gamma_{LV}=\gamma_{W}-\pi_{1}-\pi_{2},$$

In the mentioned range of surfactants concentration, the values of $\gamma_{LV}$ calculated from Equation (7) are very close to those measured (Figures S2–S5). This points out that in this range of surfactants concentration $X_{1}^{S}=\frac{\pi_{1}}{\pi_{1}+\pi_{2}}$ and $X_{2}^{S}=\frac{\pi_{2}}{\pi_{1\;}+\pi_{2}}$. The relative composition of the surfactants saturated mixed monolayer at the water–air interface can be determined using the Hua and Rosen equation which has the form [4,33]:

(8) $$\frac{{\left(X_{1}^{S}\right)}^{2}ln\left(\alpha C_{12}/X_{1}^{S}C_{1}\right)}{{\left(1-X_{1}^{S}\right)}^{2}ln\left[\left(1-\alpha \right)C_{12}/\left(1-X_{1}^{S}\right)C_{2}\right]}=1,\;$$

where α is the mole fraction of surfactant 1 (ELP) in the mixture in the bulk phase, and indices 1, 2 and 12 are related to surfactants 1, 2 and their mixture, respectively.

Taking into account the error in the calculation of mole fraction of surfactants in the mixed monolayer using Equation (8), it can be stated that in most cases the $X_{1}^{S}$ and $X_{2}^{S}$ values determined from this equation are close to those determined from $\pi_{1}$ and $\pi_{2}$ (Figure S15). Hence, the values of $X_{1}^{S}$ and $X_{2}^{S}$ obtained from $\pi_{1}$ and $\pi_{2}$, at the first approximation can be treated as a mole fraction of RH40 and ELP in the mixed monolayer at the water–air interface in the whole range of RH40 and ELP mixture concentration in the bulk phase.

In the range of ELP mixture concentration corresponding to the unsaturated mixed monolayer, i.e., from zero to 1 × 10^−6^ mol/dm^3^ in the bulk phase the mutual effect of surfactants on their adsorption was not observed and for each composition of the surfactant mixtures the mole fraction of ELP in the mixed monolayer decreases in this concentration range of the mixture in the bulk phase. However, from the beginning of the saturated mixed monolayer formation at the water–air interface the $X_{1}^{S}$ increases to the value which depends on the composition of the mixture in the bulk phase. In any case, the mole fraction of $X_{1}^{S}$ is equal to α. However, the difference between these fractions does not yet prove the existence of synergy or antagonism in the reduction in the water surface tension by the mixture of RH40 and ELP. Hua and Rosen [33] stated that the synergy and antagonistic effects in the reduction in water surface tension by the adsorption of surfactants mixture can be determined on the basis of the parameter of intermolecular interactions ($\beta^{\sigma })$. This parameter fulfills the condition [4,34]:

(9) $$\beta^{\sigma }=\frac{ln\left(\alpha C_{12}/X_{1}^{S}C_{1}\right)}{\;\;\;{\left(1-X_{1}^{S}\right)}^{2}},$$

From the calculations of the $\beta^{\sigma }$ parameter from Equation (9) it results that each value depends on the concentration and composition of the RH40 and ELP mixture as well as temperature (Figure S16). This is connected with the changes of the mixed monolayer composition at the water–air interface as a function of mixture concentration and temperature at a given constant mixture composition in the bulk phase, α. It should be noted that the values of the surfactant mole fraction in the mixed monolayer calculated from Equation (8), as mentioned above, are close to those determined on the basis of the surface tension isotherms of RH40 and ELP. The $\beta^{\sigma }$ parameter can assume both the positive and negative values (Table S1 in Supplementary Materials). For the mixture of RH40 and ELP with the ELP mole fraction equal to 0.6 the values of $\beta^{\sigma }$ are only positive and there is the almost linear dependence between $\beta^{\sigma }$ and the surface tension of solutions and temperature (Figure S16d). This indicates the synergetic effect in the water surface tension does not occur for this mixture. For the other RH40 and ELP mixtures this effect was noted but not at each temperature applied in the studies. As mentioned above, in the case of the RH40 and ELP mixture, the synergetic effect in the reduction in water surface tension results probably from the changes of the configuration of RH40 and ELP molecules in the mixed monolayer, in comparison to the individual surfactant monolayer and/or the changes of number of hydrogen bonds as a result of dehydration of surfactants tail and head.

### 2.3. Thermodynamic Parameters of Adsorption

The standard Gibbs free energy ($\Delta G_{ads}^{0}$), enthalpy ($\Delta H_{ads}^{0}$) and entropy ($\Delta S_{ads}^{0}$) of adsorption are powerful in understanding the adsorption process of the RH40 and ELP mixture at the water–air interface [4]. $\Delta G_{ads}^{0}$ informs only about the surfactants tendency to adsorb at the water–air interface. Based on this it is not possible to conclude what changes took place in the bulk phase as a result of surfactants adsorption. The $\Delta H_{ads}^{0}$ value informs us about chemical reactions which occurred during the adsorption process. In other words, based on $\Delta H_{ads}^{0}$ it can be stated whether more chemical bonds were broken or whether new ones were created. On the other hand, $\Delta S_{ads}^{0}$ informs about the structural and orientation changes of molecules in the bulk phase during the adsorption process. $\Delta S_{ads}^{0}$ is the main force of this process.

The literature reports numerous methods for determination of $\Delta G_{ads}^{0}$. Among them, those that are based on the Langmuir equation modified by de Boer are very often used. This equation has the form [35,36]:

(10) $$\frac{A^{0}}{A-A^{0}}exp\frac{A^{0}}{A-A^{0}}=\frac{C}{\varpi }exp\left(\frac{-\Delta G_{ads}^{0}}{RT}\right),$$

where A and $A^{0}$ are the areas occupied by one molecule in the monolayer and limiting one.

$\Delta G_{ads}^{0}$ can be also determined from the linear Langmuir equation [4,35]:

(11) $$\frac{C}{\Gamma }=\frac{C}{\Gamma^{max}}+\frac{a}{\Gamma^{max}},$$

where a fulfills Equation (4).

The $\Delta G_{ads}^{0}$ values were determined from Equations (10) and (3). In Equation (3) there were used the values of a established from the Szyszkowski equation and obtained from the Langmuir linear equation. Hence, for each system three values of $\Delta G_{ads}^{0}$ were obtained. There are small differences between the $\Delta G_{ads}^{0}$ values obtained using these methods (Table S2 in Supplementary Materials). There is no linear dependence between $\Delta G_{ads}^{0}$ and the composition of RH40 and ELP mixtures. The absolute values of $\Delta G_{ads}^{0}$ for RH40, ELP and their mixtures are higher than those for other nonionic surfactants. This explains why, at small concentrations of RH40, ELP and their mixtures, the reduction in water surface tension by their adsorption is higher than that of other nonionic surfactants [4,22].

It is known that $\Delta G_{ads}^{0}$ fulfills the expression [4,35]:

(12) $${\Delta G}_{ads}^{0}={\Delta H}_{ads}^{0}-T{\Delta S}_{ads}^{0},$$

Assuming that in the temperature range from 293 to 318 K the $\Delta H_{ads}^{0}$ is constant then [4,35]:

(13) $$\frac{\partial \Delta G_{ads}^{0}}{\partial T}=-\Delta S_{ads}^{0},$$

Introducing the values of $\Delta S_{ads}^{0}$ calculated from Equation (13) to Equation (12) the values of $\Delta H_{ads}^{0}$ were determined.

It follows from Table 1 that the values of $\Delta H_{ads}^{0}$ for the RH40 and ELP mixture with the mole fraction of 0.6 are close to zero. For this mixture no synergetic effect is observed in the reduction in water surface tension. For the mixture of different compositions, the positive and negative values of $\Delta H_{ads}^{0}$ were obtained. However, the absolute value of $\Delta H_{ads}^{0}$ is not large.

The nonlinear dependence between the $\Delta G_{ads}^{0}$ and composition of the RH40 and ELP mixture can result from the fact that the Gibbs free energy of RH40 and ELP mixing is different from zero. In the other words, the mixed monolayer is not ideal and the coefficients of RH40 and ELP activity are different from zero (Table S1). The coefficient of surfactants activity and next the Gibbs free energy of surfactants mixing can be determined based on the parameter of the intermolecular interactions. The coefficients of surfactants activity satisfy the condition [4]:

(14) $$lnf_{1}=\beta^{\sigma }{\left(1-X_{1}^{s}\right)}^{2},$$

and

(15) $$lnf_{2}=\beta^{\sigma }{\left(X_{1}^{s}\right)}^{2}.$$

In turn the Gibbs free energy of surfactants mixing ($G_{mix}^{E})$ can be expressed by equation [37]:

(16) $$G_{mix}^{E}=RT\left(X_{1}^{s}lnf_{1}+X_{2}^{s}lnf_{2}\right),$$

It was proved that the absolute values of $G_{mix}^{E}$ are not great. This means that there is no great difference between the ideal and real behavior of the RH40 and ELP mixtures.

## 3. Materials and Methods

For our studies the aqueous solution of Kolliphor^®^ ELP (ELP) (Cremophor^®^ ELP, Polyoxyl 35 Hydrogenated Castor oil, Polyoxyl-35 castor oil, CAS number 61791-12-6,) and Kolliphor^®^ RH 40 (RH40) (Cremophor^®^ RH 40, macrogolglycerol hydroxystearate, PEG-40 castor oil, Polyoxyl 40 hydrogenated castor oil, CAS number 61788-85-0,) mixture were applied. RH40 and ELP were purchased form Sigma-Aldrich (St. Louis, MO, USA) and used without additional purification. The solution concentration was in the range from 0 to the values significantly higher than the critical micelle concentration (CMC) of RH40 and ELP that is from 0 to 0.01 M (mol/dm^3^) and the ELP mole fraction in the surfactant mixtures in the bulk phase, *α*, was equal to 0.2, 0.4, 0.6 and 0.8. The water used for the solutions preparation was doubly distilled and deionized (Destamat) the surface tension of which changed from 72.8 to 68.7 mN/m in the temperature range from 293 to 318 K.

The surface tension ($\gamma_{LV}$) measurements of the aqueous solution of the RH40 and ELP mixture were made in the temperature range from 293 to 318 K using the Krüss K100 tensiometer according to the platinum ring tensiometer method (du Nouy’s method) calibrated before the measurements. The calibration was made only at 293 K using water and methanol whose surface tension at this temperature was equal to 72.8 and 22.5 mN/m, respectively. The surface tension measurements for each concentration and composition of the aqueous solution of RH40 and ELP mixtures were repeated at least ten times. The standard deviation of the results obtained from the measurements was ± 0.1 mN/m, and the uncertainty was in the range from 0.3% to 0.9%.

## 4. Conclusions

Based on the results obtained from the surface tension measurements of the aqueous solution of RH40 and ELP mixtures and their analysis many conclusions can be drawn.

In the small concentration range of RH40 and ELP as well as their mixture in the aqueous solution the greater reduction in water surface tension takes place as a result of the adsorption at the water–air interface that due to the adsorption of other nonionic surfactants. However, the minimal surface tension of the aqueous solution of RH40 and ELP and their mixtures which can be obtained is close to that of TX165.

There is no linear dependence between the surface tension of RH40, ELP mixtures and their composition. The isotherm of the surface tension of RH40 and ELP, as well as their mixture, can be precisely described by the exponential function of the second order. It is possible to describe the isotherm of the surface tension by the numerical solution of the Szyszkowski equation. The maximal Gibbs surface excess concentration obtained solving the Szyszkowski equation against the surface tension is comparable to that determined from the equation of the Gibbs isotherm of adsorption.

The surface tension of the aqueous solution of RH40 and ELP mixtures can be predicted using the Fainerman and Miller equation in the range of mixtures concentration from zero to CMC. In some cases, it is possible to predict the changes of the surface tension of the aqueous solution of RH40 and ELP mixtures in the whole studied range of concentration.

The synergetic effect of the RH40 and ELP mixtures in the reduction in the water surface tension but not at all their composition was found based on the intermolecular interactions parameter.

The composition of the RH40 and ELP mixed monolayer at the water–air interface can be predicted from the contribution of a particular surfactant to the reduction in water surface tension.

The calculated values of the mole fraction of RH40 and ELP in the mixed monolayer at the water–air interface, on the basis of their aqueous solution surface tension isotherms, are in most cases close to those determined based on the Rosen and Hua concept.

The values of the Gibbs free energy of adsorption for a given system calculated from the constant in the Szyszkowski equation are close to those calculated from the Langmuir equation modified by de Boer and the linear Langmuir equation.

The absolute values of the Gibbs free energy of adsorption for the studied systems are higher than those for Triton’s.

The standard enthalpy of adsorption assumes negative and positive values depending on the composition of the RH40 and ELP mixture, but the absolute values of enthalpy do not differ significantly from zero.

## Figures and Tables

**Table 1:** The values of the $\Delta H_{ads}^{0}$ and $\Delta S_{ads}^{0}$ for the RH40 and ELP as well as their binary mixtures calculated from Equations (12) and (13), respectively.

$\Delta S_{ads}^{0}$ [kJ/molK]						
RH40		α = 0.2	α = 0.4	α = 0.6	α = 0.8	ELP
−0.172		−0.178	−0.191	−0.146	−0.159	−0.161
$\Delta H_{ads}^{0}$ [kJ/mol]						
T [K]	RH40	α = 0.2	α = 0.4	α = 0.6	α = 0.8	ELP
293	3.344	5.034	8.984	−3.262	−0.197	0.857
298	3.403	5.123	9.140	−2.983	−0.201	0.871
303	3.473	5.221	9.305	−3.034	−0.196	0.896
308	3.292	5.069	9.101	−3.084	−0.200	0.910
313	3.352	5.037	8.977	−3.125	−0.195	0.845
318	3.411	5.116	9.123	−3.166	−0.199	0.869

## Data Availability

The data presented in this study are available in Supplementary Materials.

## Supplementary Materials

The following are available online, Scheme S1: The structure of the main components of ELP (a) and RH40 (b); Table S1: The values of the mole fractions of ELP ($X_{1}^{S}$) and RH40 ($X_{2}^{S}$) in the mixed monolayer at the water–air interface, parameter of intermolecular interactions ($\beta^{\sigma })$, activity coefficient of ELP ($f_{1}$) and RH40 ($f_{2}$) as well as and Gibbs free energy of surfactants mixing ($G_{mix}^{E})$ (kJ/mol) for the mixtures at the mole fraction of ELP in the bulk phase, $\alpha_{}$ equal to 0.2 (a), 0.4 (b), 0.6 (c) and 0.8 (d). There is also the condition for synergism or antagonism existence ($\ln(C_{1/}C_{2})$) [4]; Table S2: The values of the standard Gibbs free energy of adsorption ($\Delta G_{ads}^{0}$) calculated from Equations (3), (10) and (11); Figure S1: A plot of the surface tension ($\gamma_{LV}$) of the aqueous solutions of RH40 vs. the logarithm of its concentration (logC) at different temperatures equal to 293 (a), 298 (b), 303 (c), 308 (d), 313 (e) and 318 K (f). Points 1 correspond to the measured values, curves 2 and 3 correspond to the values calculated from the Szyszkowski equation (Equation (2)) and the exponential function of the second order (Equation (1)), respectively; Figure S2: A plot of the surface tension ($\gamma_{LV}$) of the aqueous solutions of RH40 and ELP mixtures at the mole fraction of ELP in the bulk phase equal to 0.2 vs. the logarithm of their concentration ($\log C_{12}$) at different temperatures equal to 293 (a), 298 (b), 303 (c), 308 (d), 313 (e) and 318 K (f). Points 1 correspond to the measured values, curves 2–6 correspond to the values calculated from the Szyszkowski equation (Equation (2)), exponential function of the second order (Equation (1)), Fainerman and Miller equation (Equation (5)), Equation (7) and Equation (4), respectively; Figure S3: A plot of the surface tension ($\gamma_{LV}$) of the aqueous solutions of RH40 and ELP mixtures at the mole fraction of ELP in the bulk phase equal to 0.4 vs. the logarithm of their concentration ($\log C_{12}$) at different temperatures equal to 293 (a), 298 (b), 303 (c), 308 (d), 313 (e) and 318 K (f). Points 1 correspond to the measured values, curves 2–6 correspond to the values calculated from the Szyszkowski equation (Equation (2)), exponential function of the second order (Equation (1)), Fainerman and Miller equation (Equation (5)), Equation (7) and Equation (4), respectively; Figure S4: A plot of the surface tension ($\gamma_{LV}$) of the aqueous solutions of RH40 and ELP mixtures at the mole fraction of ELP in the bulk phase equal to 0.6 vs. the logarithm of their concentration ($\log C_{12}$) at different temperatures equal to 293 (a), 298 (b), 303 (c), 308 (d), 313 (e) and 318 K (f). Points 1 correspond to the measured values, curves 2–6 correspond to the values calculated from the Szyszkowski equation (Equation (2)), exponential function of the second order (Equation (1)), Fainerman and Miller equation (Equation (5)), Equation (7) and Equation (4), respectively; Figure S5: A plot of the surface tension ($\gamma_{LV}$) of the aqueous solutions of RH40 and ELP mixtures at the mole fraction of ELP in the bulk phase equal to 0.8 vs. the logarithm of their concentration ($\log C_{12}$) at different temperatures equal to 293 (a), 298 (b), 303 (c), 308 (d), 313 (e) and 318 K (f). Points 1 correspond to the measured values, curves 2–6 correspond to the values calculated from the Szyszkowski equation (Equation (2)), exponential function of the second order (Equation (1)), Fainerman and Miller equation (Equation (5)), Equation (7) and Equation (4), respectively; Figure S6: A plot of the surface tension ($\gamma_{LV}$) of the aqueous solutions of ELP vs. the logarithm of its concentration (logC) at different temperatures equal to 293 (a), 298 (b), 303 (c), 308 (d), 313 (e) and 318 K (f). Points 1 correspond to the measured values, curves 2 and 3 correspond to the values calculated from the Szyszkowski equation (Equation (2)) and the exponential function of the second order (Equation (1)), respectively; Figure S7: A plot of the values of constant $y_{0}$ in Equation (1) (curve 1) and the minimal surface tension of aqueous solution ($\gamma_{LV}^{min}$) (curve 2) vs. the temperature (T) for the RH40 and ELP mixtures at the mole fraction of ELP in the bulk phase equal to 0 (RH40 (a)), 0.2 (b), 0.4 (c), 0.6 (d), 0.8 (e) and 1 (ELP (f)), respectively; Figure S8: A plot of the values of constant $y_{0}$ in Equation (1) for studied surfactant mixtures vs. the mole fraction of ELP in the bulk phase (α) at the temperatures equal to 293 (curve 1), 298 (curve 2), 303 (curve 3), 308 (curve 4), 313 (curve 5) and 318 K (curve 6), respectively; Figure S9: A plot of the values of constant $A_{1}$ in Equation (1) for studied surfactant mixtures vs. the mole fraction of ELP in the bulk phase (α) at the temperatures equal to 293 (curve 1), 298 (curve 2), 303 (curve 3), 308 (curve 4), 313 (curve 5) and 318 K (curve 6), respectively; Figure S10: A plot of the values of constant $A_{2}$ in Equation (1) for studied surfactant mixtures vs. the mole fraction of ELP in the bulk phase (α) at the temperatures equal to 293 (curve 1), 298 (curve 2), 303 (curve 3), 308 (curve 4), 313 (curve 5) and 318 K (curve 6), respectively; Figure S11: A plot of the values of constant $t_{1}$ in Equation (1) for studied surfactant mixtures vs. the mole fraction of ELP in the bulk phase (α) at the temperatures equal to 293 (curve 1), 298 (curve 2), 303 (curve 3), 308 (curve 4), 313 (curve 5) and 318 K (curve 6), respectively; Figure S12: A plot of the values of constant $t_{2}$ in Equation (1) for studied surfactant mixtures vs. the mole fraction of ELP in the bulk phase (α) at the temperatures equal to 293 (curve 1), 298 (curve 2), 303 (curve 3), 308 (curve 4), 313 (curve 5) and 318 K (curve 6), respectively; Figure S13: The values of $\Gamma^{max}$ calculated from Equations (6) and (2) for studied surfactant mixtures at the mole fraction of ELP in the bulk phase (α) equal to 0, 0.2, 0.4, 0.6, 0.8 and 1 at different temperatures equal to 293 (a), 298 (b), 303 (c), 308 (d), 313 (e) and 318 K (f), respectively; Figure S14: A plot of the values of Gibbs surface excess concentration (Γ) vs. logarithm of the concentration in the bulk phase (*C* or *C*_12_) for the RH40 and ELP mixtures at the mole fraction of ELP in the bulk phase equal to 0 (RH40 (a)), 0.2 (b), 0.4 (c), 0.6 (d), 0.8 (e) and 1 (ELP (f)), respectively; Figure S15: The values of the mole fraction of ELP in the mixed monolayer at the water air interface ($X_{1}^{S}$) calculated from the relationship: $X_{1}^{S}=\frac{\pi_{1}}{\pi_{1}+\pi_{2}}\;$(bars 1) and from Equation (8) (bars 2) and temperature range 293-318K for mixtures at the mole fraction of ELP in the bulk phase (α) equal to 0.2 (a), 0.4 (b), 0.6 (c) and 0.8 (d). Figure S16: A plot of the values of parameter of intermolecular interactions, $\beta^{\sigma },$ for studied binary surfactant mixtures (a) at the mole fraction of ELP in the bulk phase *α*, equal to 0.2, 0.4, 0.6, 0.8 and T = 293K vs. surface tension, ($\gamma_{LV}$), (b) at the surface tension, ($\gamma_{LV}$), equal to 65, 55, 45 mN/m and T = 293K vs. mole fraction of ELP in the bulk phase *α,* (c) at the mole fraction of ELP in the bulk phase *α*, equal to 0.2, 0.4, 0.6, 0.8 and $\gamma_{LV}\;$= 65 mN/m vs. temperature, T, (d) at the mole fraction of ELP in the bulk phase *α*, equal to 0.6 and $\gamma_{LV}\;$= 65, 55, 45 mN/m vs. temperature, T.

## Sample Availability

Samples of the compounds are not available from the authors.
