# Supplementary material for: Adsorption Properties and Composition of Binary Kolliphor Mixtures at the Water–Air Interface at Different Temperatures
Source: Molecules. 2022 Jan 27;27(3):877. doi: 10.3390/molecules27030877 (PMC8839182; doi:10.3390/molecules27030877)
Supplement: Supplementary file 1 [file molecules-27-00877-s001.zip › molecules-1542633-supplementary.pdf]

Supplementary Materials

Adsorption properties and composition of binary Kolliphor mixtures at the water-air interface at different temperatures

Magdalena Szaniawska[1], Katarzyna Szymczyk[1], Anna Zdziennicka[1], and Bronisław Jańczuk[1,*]

[1] Department of Interfacial Phenomena, Institute of Chemical Sciences, Faculty of Chemistry, Maria Curie-Skłodowska University in Lublin, Maria Curie-Skłodowska Sq. 3, 20-031 Lublin, Poland;
* Correspondence: bronislaw.janczuk@poczta.umcs.lublin.pl; Tel.: +48-81-537-56-49

a)

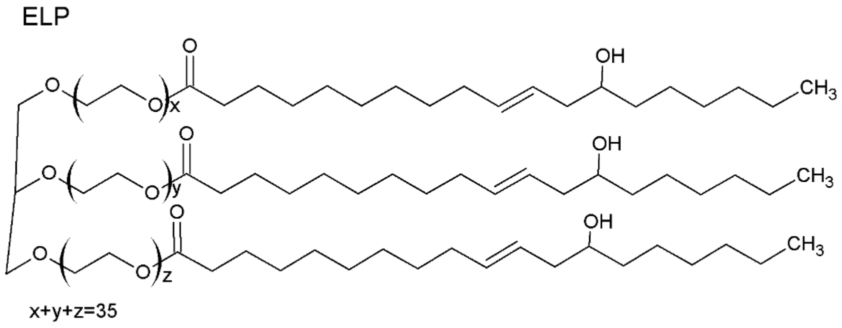

b)

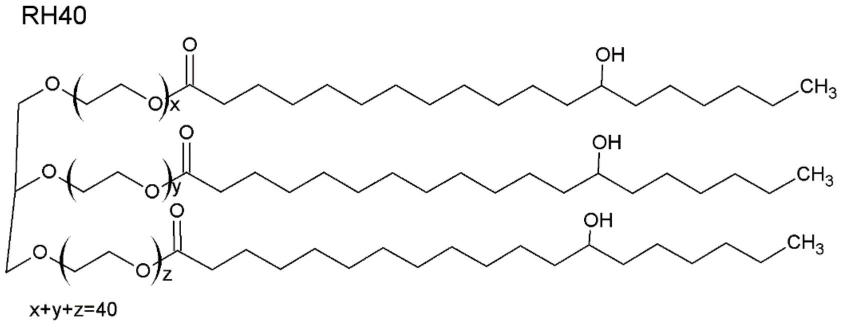

Scheme S1. The structure of the main components of ELP (a) and RH40 (b).

Table S1. The values of the mole fractions of ELP (X_1^S) and RH40 (X_2^S) in the mixed monolayer at the water-air interface, parameter of intermolecular interactions (β^σ), activity coefficient of ELP (f_1) and RH40 (f_2) as well as and Gibbs free energy of surfactants mixing (G_{mix}^E) (kJ/mol) for the mixtures at the mole fraction of ELP in the bulk phase, α equal to 0.2 (a), 0.4 (b), 0.6 (c) and 0.8 (d). There is also the condition for synergism or antagonism existence ($\ln(C_1/C_2)$) [4].

a)

			α ELP = 0.2, γ_{LV} = 65 mN/m				
T [K]	X_1^S	X_2^S	β^σ	$\ln(C_1/C_2)$	f_1	f_2	G_{mix}^E
293	0.2708	0.7292	−1.7006	0.3838	0.4048	0.8828	−0.8180
298	0.2603	0.7397	−1.6267	0.4380	0.4106	0.8956	−0.7760
303	0.2365	0.7635	−1.4850	0.4960	0.4208	0.9203	−0.6755
308	0.2405	0.7595	−1.4010	0.5388	0.4456	0.9222	−0.6552
313	0.2518	0.7482	−1.2870	0.3152	0.4865	0.9216	−0.6310
318	0.2673	0.7327	−1.2858	0.2217	0.5014	0.9122	−0.6658
			α ELP = 0.2, γ_{LV} = 55 mN/m				
293	0.2722	0.7278	−0.8054	0.2215	0.6527	0.9421	−0.3887
298	0.2610	0.7390	−0.2582	0.3000	0.8685	0.9826	−0.1234
303	0.2400	0.7600	0.3737	0.3797	1.2409	1.0218	0.1717
308	0.2510	0.7490	0.0917	0.4241	1.0528	1.0058	0.0441
313	0.2568	0.7432	−1.2022	0.2597	0.5148	0.9238	−0.5971
318	0.2856	0.7144	−1.4203	0.1393	0.4844	0.8906	−0.7662
			α ELP = 0.2, γ_{LV} = 45 mN/m				
293	0.27682	0.72318	0.27178	0.5473	1.1527	1.0210	0.1325
298	0.2680	0.7320	0.72404	0.3283	1.4740	1.0534	0.3519
303	0.2500	0.7500	2.42876	0.1432	3.9203	1.1639	1.1472
308	0.2590	0.7410	−0.20318	0.0836	0.8944	0.9865	−0.0999
313	0.3200	0.6800	−1.82648	0.1750	0.4297	0.8294	−1.0343
318	0.34357	0.65643	−1.77151	0.1837	0.4661	0.8113	−1.0563

b)

			α ELP = 0.4, γ_{LV} = 65 mN/m				
T [K]	X_1^S	X_2^S	β^σ	$\ln(C_1/C_2)$	f_1	f_2	G_{mix}^E
293	0.3267	0.6733	−0.1910	0.3838	0.9171	0.9798	−0.1023
298	0.2837	0.7163	0.1910	0.4380	1.1029	1.0155	0.0962
303	0.2935	0.7065	−0.0529	0.4960	0.9739	0.9955	−0.0276
308	0.3312	0.6689	−0.7090	0.5388	0.7282	0.9252	−0.4021
313	0.3854	0.6146	−1.7359	0.3152	0.5191	0.7727	−1.0700
318	0.4333	0.5667	−2.6775	0.2217	0.4232	0.6050	−1.7382
			α ELP = 0.4, γ_{LV} = 55 mN/m				
293	0.3801	0.6199	−0.5749	0.2215	0.8018	0.9203	−0.3300
298	0.3532	0.6468	−0.3420	0.3000	0.8667	0.9582	−0.1936
303	0.3334	0.6666	−0.2679	0.3797	0.8878	0.9707	−0.1500
308	0.3397	0.6603	−0.5233	0.4241	0.7960	0.9414	−0.3006
313	0.3950	0.6050	−1.1453	0.2597	0.6576	0.8363	−0.7122
318	0.4287	0.5713	−1.8049	0.1393	0.5548	0.7177	−1.1687
			α ELP = 0.4, γ_{LV} = 45 mN/m				
293	0.5260	0.4740	−0.7231	0.5473	0.8501	0.8187	−0.4392
298	0.4873	0.5127	−0.7825	0.3283	0.8141	0.8304	−0.4844
303	0.4536	0.5464	−0.8350	0.1432	0.7793	0.8422	−0.5213
308	0.4437	0.5563	−0.8497	0.0836	0.7688	0.8460	−0.5370
313	0.4630	0.5370	−1.1080	0.1750	0.7265	0.7886	−0.7169
318	0.4664	0.5336	−1.2825	0.1837	0.6941	0.7566	−0.8438

c)

T [K]	X_1^S	X_2^S	β^σ	$\ln(C_1/C_2)$	f_1	f_2	G_{mix}^E
			α ELP = 0.6, γ_{LV} = 65 mN/m				
293	0.5060	0.4940	0.2101	0.3838	1.0526	1.0553	0.1279
298	0.4908	0.5092	0.2404	0.438	1.0643	1.0596	0.1489
303	0.4733	0.5267	0.3271	0.4960	1.0950	1.0760	0.2054
308	0.4679	0.5321	0.3505	0.5388	1.1043	1.0798	0.2235
313	0.5201	0.4799	0.3944	0.3152	1.0951	1.1126	0.2562
318	0.5583	0.4417	0.4225	0.2217	1.0859	1.1407	0.2755
			α ELP = 0.6, γ_{LV} = 55 mN/m				
293	0.6090	0.3910	1.1888	0.2215	1.1993	1.5541	0.6896
298	0.5933	0.4067	1.4586	0.3000	1.2729	1.6710	0.8720
303	0.5660	0.4340	1.6555	0.3797	1.3659	1.6995	1.0244
308	0.5580	0.4420	1.7510	0.4241	1.4079	1.7250	1.1059
313	0.6401	0.3599	1.8977	0.2597	1.2787	2.1761	1.1377
318	0.7400	0.2600	2.0000	0.1393	1.1448	2.9898	1.0174
			α ELP = 0.6, γ_{LV} = 45 mN/m				
293	0.6400	0.3600	1.5235	0.5473	1.2183	1.8664	0.8551
298	0.6300	0.3700	1.8585	0.3283	1.2897	2.0910	1.0553
303	0.6400	0.3600	1.9591	0.1432	1.2890	2.2310	1.1371
308	0.6500	0.3500	2.0930	0.0836	1.2923	2.4213	1.2193
313	0.7000	0.3000	2.3117	0.1750	1.2313	3.1041	1.2633
318	0.8000	0.2000	2.3452	0.1837	1.0983	4.4859	0.9921

d)

T [K]	X_1^S	X_2^S	β^σ	$\ln(C_1/C_2)$	f_1	f_2	G_{mix}^E
			α ELP = 0.8, γ_{LV} = 65 mN/m				
293	0.6783	0.3217	−0.7193	0.3838	0.9283	0.7182	−0.3823
298	0.6926	0.3074	−0.3532	0.4380	0.9672	0.8442	−0.1863
303	0.7063	0.2937	−0.0337	0.4960	0.9971	0.9833	−0.0176
308	0.6807	0.3193	0.2259	0.5388	1.0233	1.1103	0.1257
313	0.7098	0.2902	0.5465	0.3152	1.0471	1.3170	0.2929
318	0.8291	0.1709	0.6282	0.2217	1.0185	1.5400	0.2353
			α ELP = 0.8, γ_{LV} = 55 mN/m				
293	0.6831	0.3169	−1.0828	0.2215	0.8970	0.6033	−0.5710
298	0.7016	0.2984	−0.8880	0.3000	0.9240	0.6459	−0.4606
303	0.6893	0.3107	−0.5618	0.3797	0.9472	0.7657	−0.3031
308	0.6947	0.3053	−0.3522	0.4241	0.9677	0.8437	−0.1912
313	0.7883	0.2117	0.3289	0.2597	1.0148	1.2267	0.1428
318	0.9298	0.0702	1.5557	0.1393	1.0077	3.8385	0.2683
			α ELP = 0.8, γ_{LV} = 45 mN/m				
293	0.7063	0.2938	−2.5609	0.5473	0.8017	0.2788	−1.2942
298	0.6929	0.3071	−2.3356	0.3283	0.8023	0.3258	−1.2314
303	0.6925	0.3075	−1.8596	0.1432	0.8388	0.4099	−0.9975
308	0.7251	0.2749	−1.1108	0.0836	0.9195	0.5577	−0.5670
313	0.7627	0.2374	−0.7509	0.175	0.9586	0.6461	−0.3537
318	0.8254	0.1746	−0.0268	0.1837	0.9992	0.9819	−0.0102

Table S2. The values of the standard Gibbs free energy of adsorption (ΔG_{ads}^{0}) calculated from Eqs. (3), (10) and (11).

Temperature [K]	ΔG_{ads}^{0} [kJ/mol]					
	RH40	$\alpha = 0.2$	$\alpha = 0.4$	$\alpha = 0.6$	$\alpha = 0.8$	ELP
			Eq. (3)			
293	−47.02	−47.02	−47.02	−45.99	−46.81	−46.29
298	−47.82	−47.82	−47.82	−46.44	−47.61	−47.08
303	−48.61	−48.61	−48.61	−47.22	−48.40	−47.86
308	−49.65	−49.65	−49.77	−48.00	−49.20	−48.65
313	−50.45	−50.57	−50.85	−48.77	−49.99	−49.52
318	−51.25	−51.38	−51.66	−49.54	−50.79	−50.30
			Eq. (10)			
293	−46.12	−45.41	−45.90	−45.03	−44.86	−45.00
298	−46.86	−47.08	−46.68	−45.81	−45.81	−45.72
303	−47.75	−48.06	−47.36	−46.38	−46.66	−46.53
308	−48.46	−49.19	−48.50	−47.23	−47.41	−47.05
313	−49.03	−49.76	−50.04	−47.68	−47.89	−48.33
318	−49.49	−50.90	−51.19	−48.32	−48.39	−49.14
			Eq. (11)			
293	−49.20	−48.14	−48.36	−48.21	−46.88	−47.68
298	−49.96	−51.11	−49.18	−49.15	−48.03	−48.62
303	−50.89	−52.07	−49.99	−49.85	−48.88	−49.55
308	−51.65	−53.23	−51.36	−50.85	−49.73	−50.03
313	−52.31	−53.39	−53.15	−51.38	−50.27	−51.12
318	−52.82	−54.72	−54.57	−52.01	−50.82	−52.37

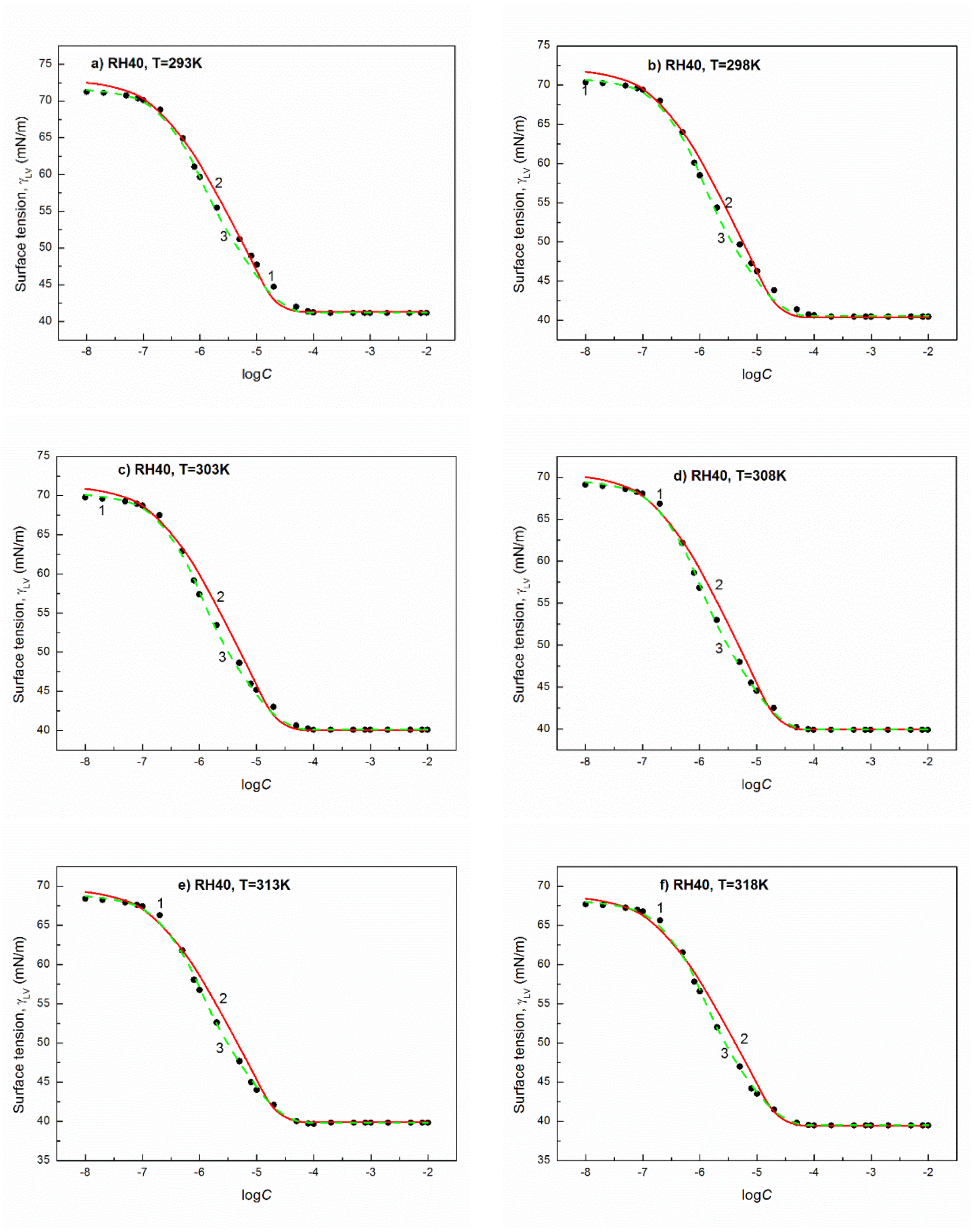

Figure S1. A plot of the surface tension (γ_{LV}) of the aqueous solutions of RH40 vs. the logarithm of its concentration (log$C$) at different temperatures equal to 293 (a), 298 (b), 303 (c), 308 (d), 313 (e) and 318 K (f). Points 1 correspond to the measured values, curves 2 and 3 correspond to the values calculated from the Szyszkowski equation (Eq. (2)) and the exponential function of the second order (Eq. (1)), respectively.

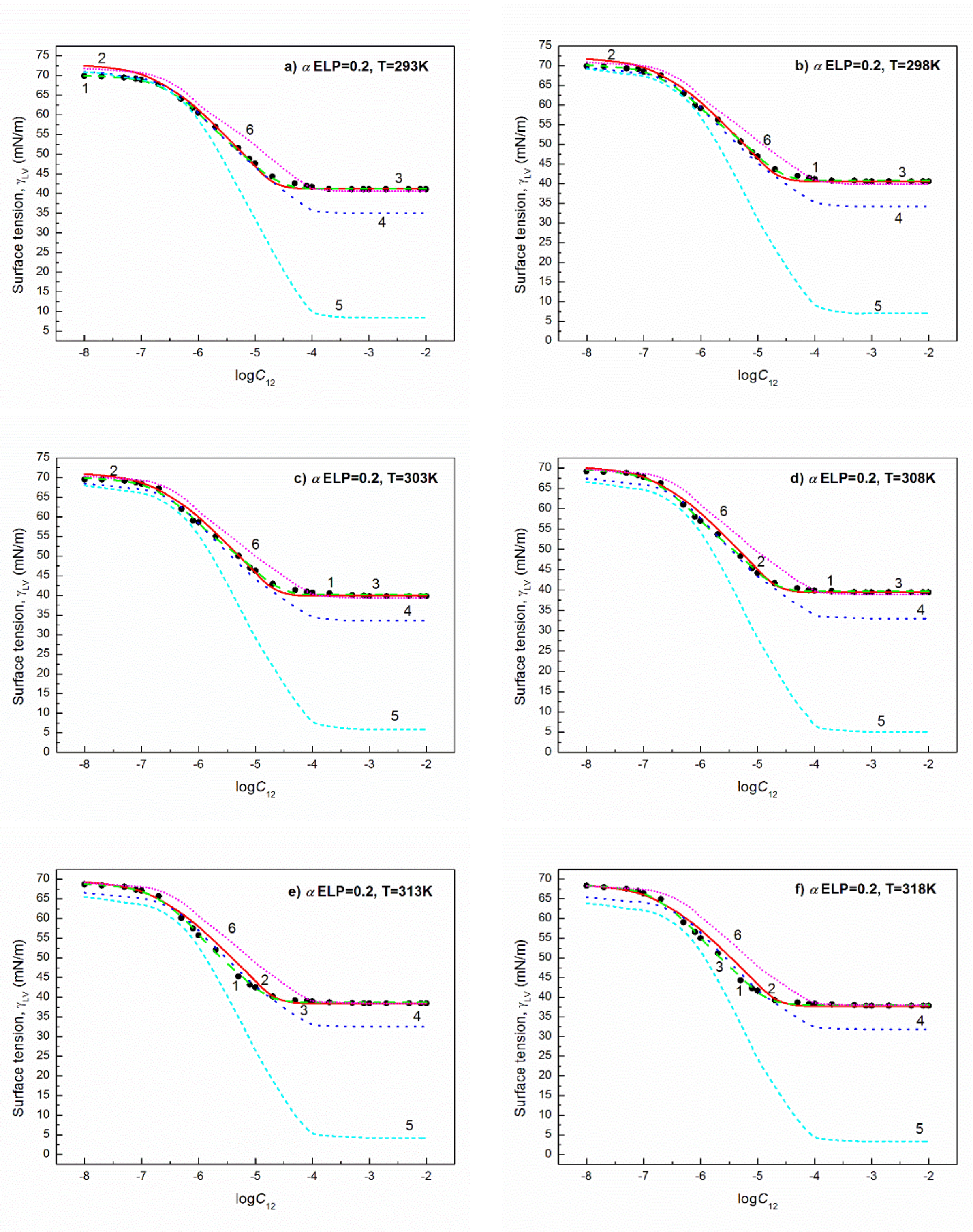

Figure S2. A plot of the surface tension (γ_{LV}) of the aqueous solutions of RH40 and ELP mixtures at the mole fraction of ELP in the bulk phase equal to 0.2 vs. the logarithm of their concentration (logC_{12}) at different temperatures equal to 293 (a), 298 (b), 303 (c), 308 (d), 313 (e) and 318 K (f). Points 1 correspond to the measured values, curves 2 – 6 correspond to the values calculated from the Szyszkowski equation (Eq. (2)), exponential function of the second order (Eq. (1)), Fainerman and Miller equation (Eq. (5)), Eq. (7) and Eq. (4), respectively.

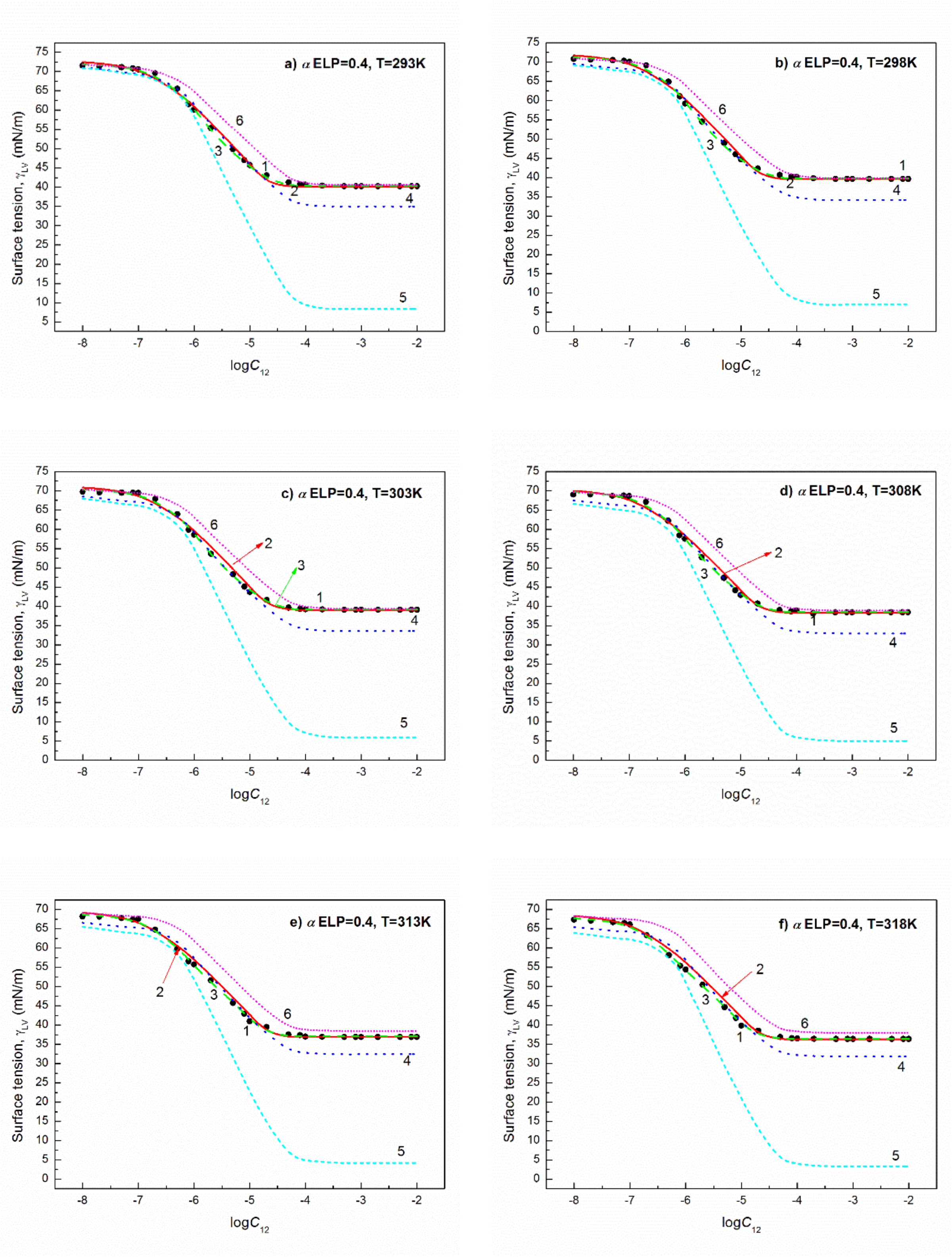

Figure S3. A plot of the surface tension (γ_{LV}) of the aqueous solutions of RH40 and ELP mixtures at the mole fraction of ELP in the bulk phase equal to 0.4 vs. the logarithm of their concentration ($\log C_{12}$) at different temperatures equal to 293 (a), 298 (b), 303 (c), 308 (d), 313 (e) and 318 K (f). Points 1 correspond to the measured values, curves 2 – 6 correspond to the values calculated from the Szyszkowski equation (Eq. (2)), exponential function of the second order (Eq. (1)), Fainerman and Miller equation (Eq. (5)), Eq. (7) and Eq. (4), respectively.

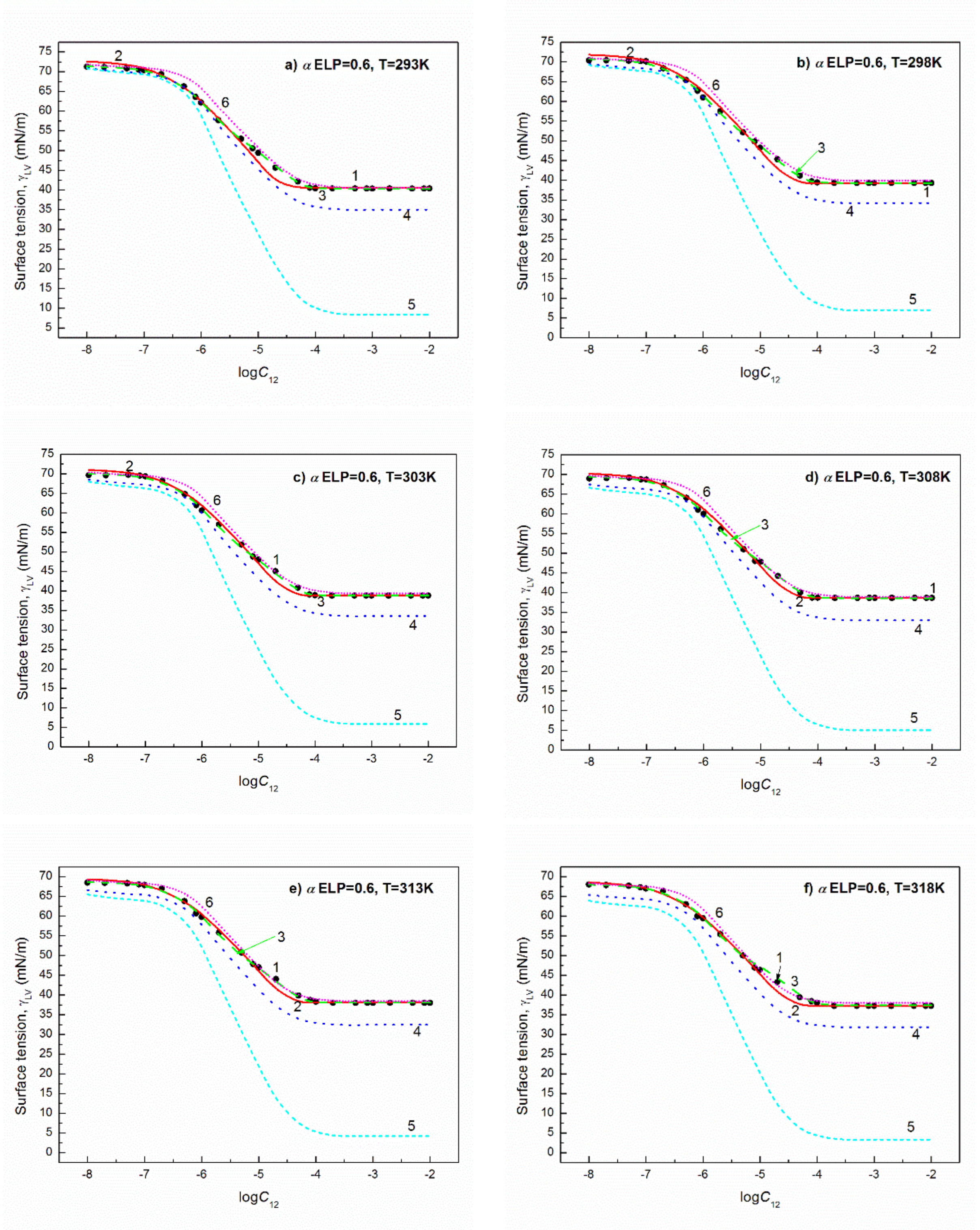

Figure S4. A plot of the surface tension (γ_{LV}) of the aqueous solutions of RH40 and ELP mixtures at the mole fraction of ELP in the bulk phase equal to 0.6 vs. the logarithm of their concentration ($\log C_{12}$) at different temperatures equal to 293 (a), 298 (b), 303 (c), 308 (d), 313 (e) and 318 K (f). Points 1 correspond to the measured values, curves 2 – 6 correspond to the values calculated from the Szyszkowski equation (Eq. (2)), exponential function of the second order (Eq. (1)), Fainerman and Miller equation (Eq. (5)), Eq. (7) and Eq. (4), respectively.

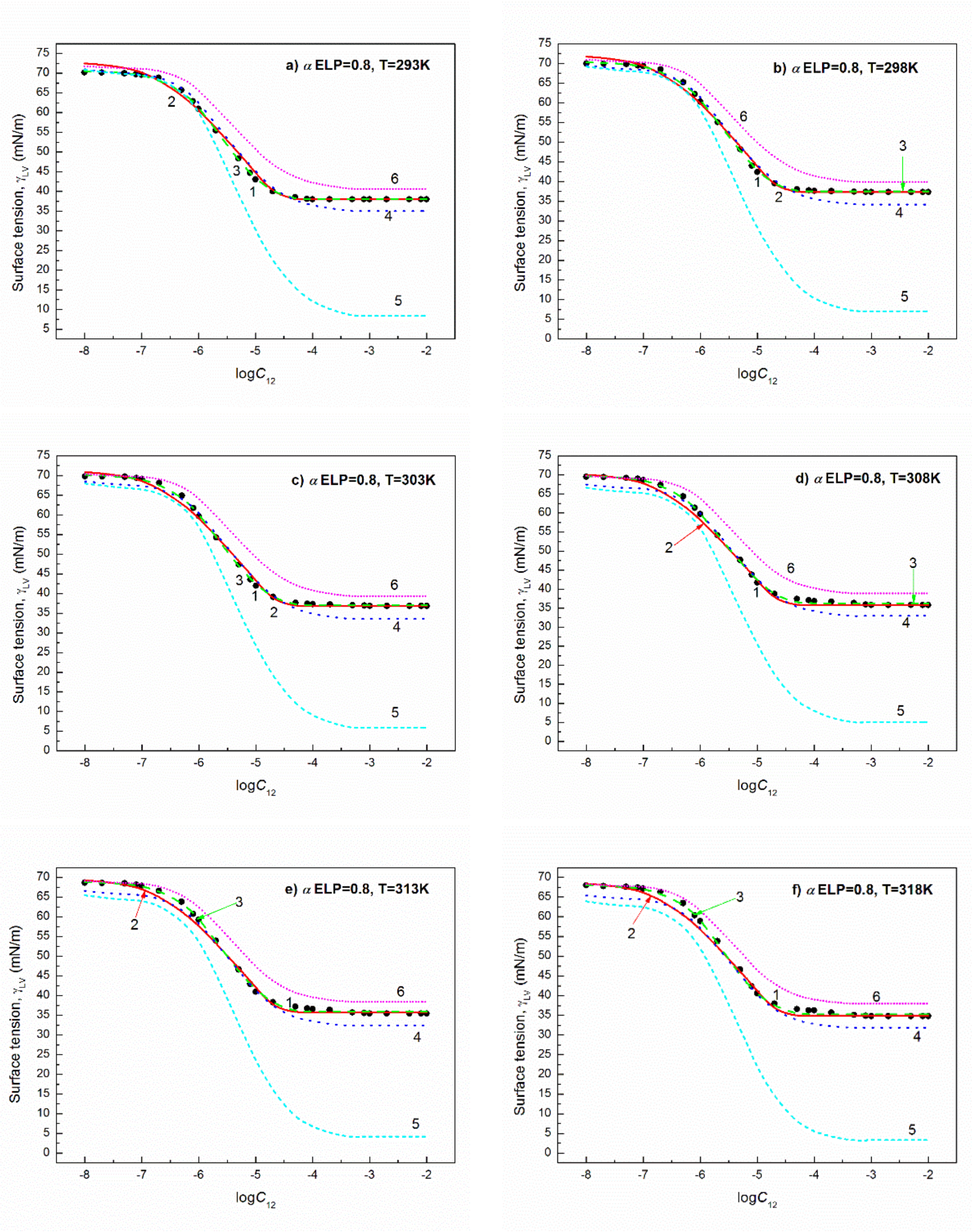

Figure S5. A plot of the surface tension (γ_{LV}) of the aqueous solutions of RH40 and ELP mixtures at the mole fraction of ELP in the bulk phase equal to 0.8 vs. the logarithm of their concentration (logC_{12}) at different temperatures equal to 293 (a), 298 (b), 303 (c), 308 (d), 313 (e) and 318 K (f). Points 1 correspond to the measured values, curves 2 – 6 correspond to the values calculated from the Szyszkowski equation (Eq. (2)), exponential function of the second order (Eq. (1)), Fainerman and Miller equation (Eq. (5)), Eq. (7) and Eq. (4), respectively.

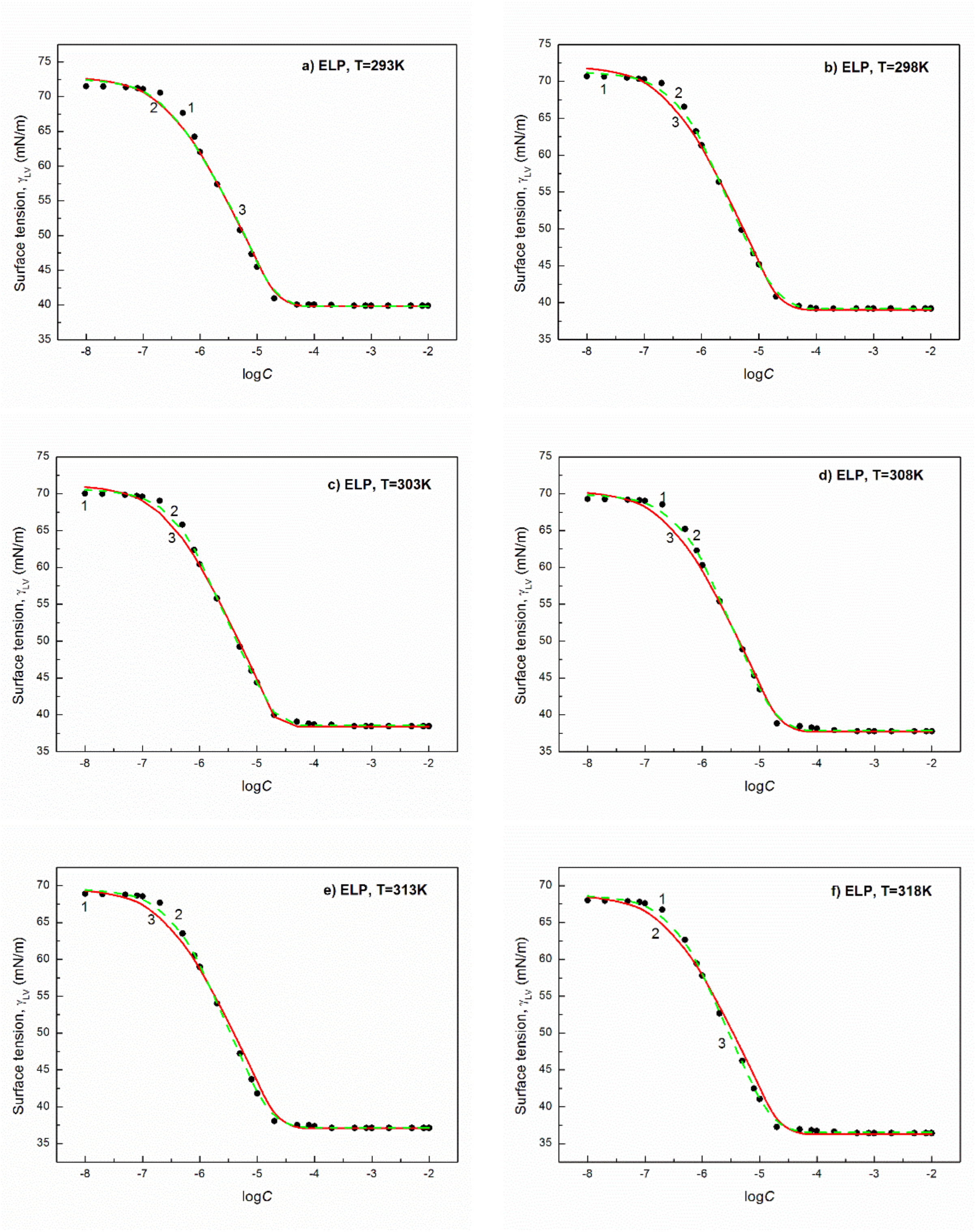

Figure S6. A plot of the surface tension (γ_{LV}) of the aqueous solutions of ELP vs. the logarithm of its concentration ($logC$) at different temperatures equal to 293 (a), 298 (b), 303 (c), 308 (d), 313 (e) and 318 K (f). Points 1 correspond to the measured values, curves 2 and 3 correspond to the values calculated from the Szyszkowski equation (Eq. (2)) and the exponential function of the second order (Eq. (1)), respectively.

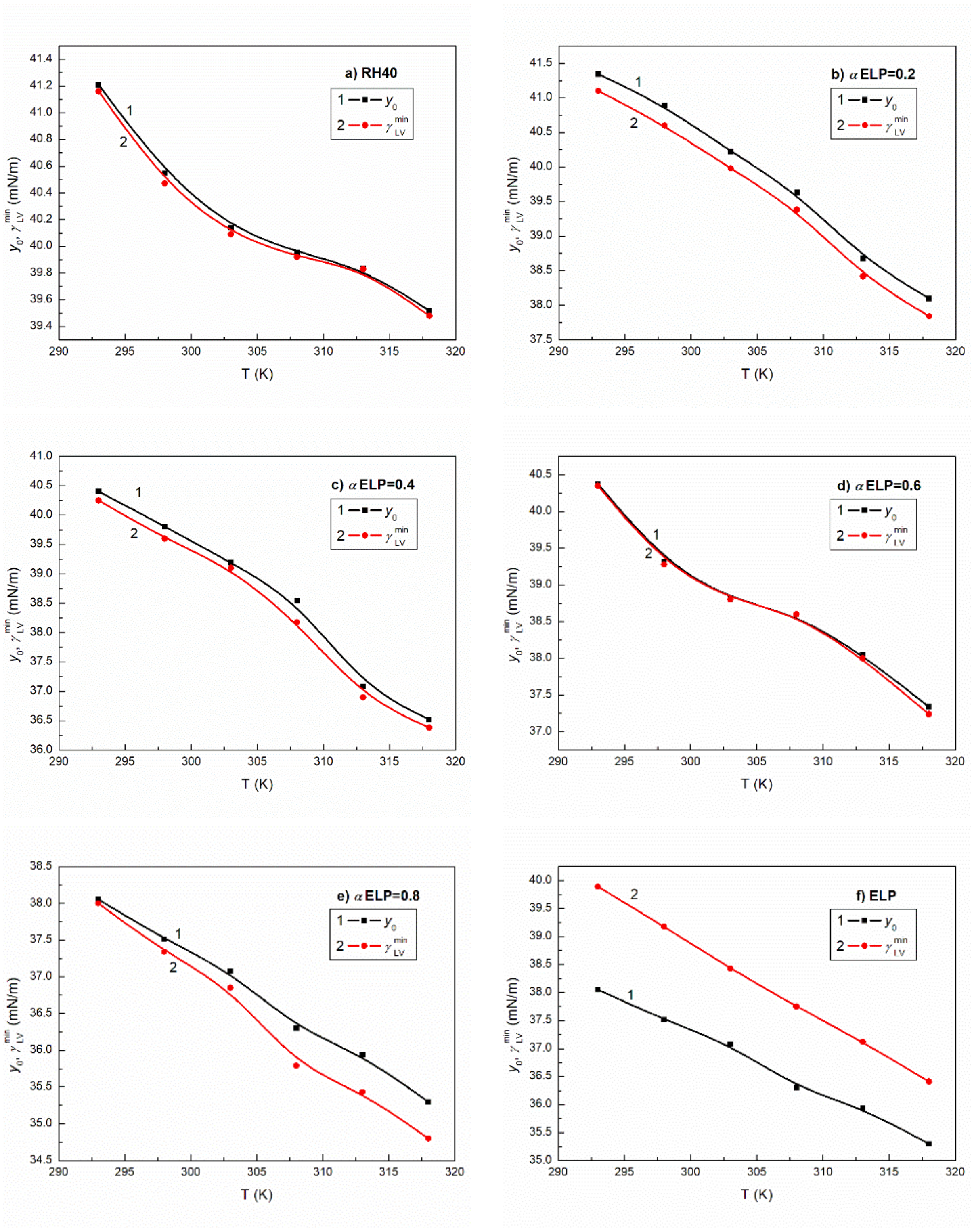

Figure S7. A plot of the values of constant y_0 in Eq. (1) (curve 1) and the minimal surface tension of aqueous solution (γ_{LV}^{min}) (curve 2) vs. the temperature (T) for the RH40 and ELP mixtures at the mole fraction of ELP in the bulk phase equal to 0 (RH40 (a)), 0.2 (b), 0.4 (c), 0.6 (d), 0.8 (e) and 1 (ELP (f)), respectively.

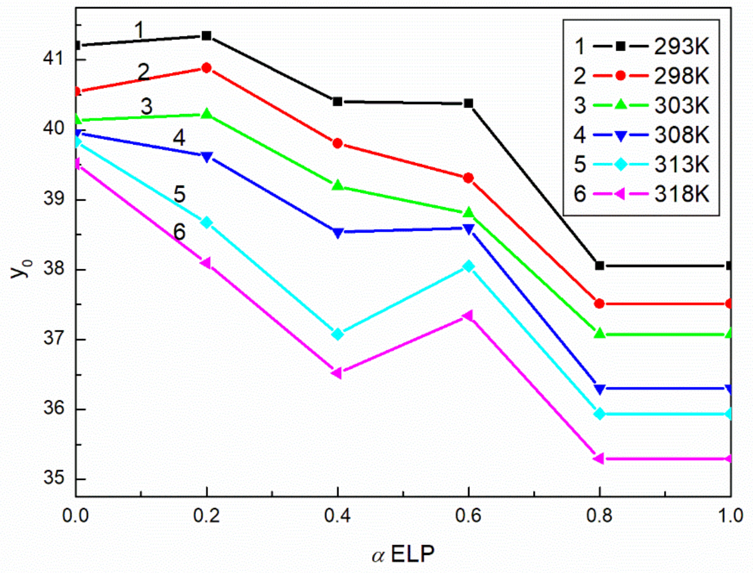

Figure S8. A plot of the values of constant y_0 in Eq. (1) for studied surfactant mixtures vs. the mole fraction of ELP in the bulk phase (α) at the temperatures equal to 293 (curve 1), 298 (curve 2), 303 (curve 3), 308 (curve 4), 313 (curve 5) and 318 K (curve 6), respectively.

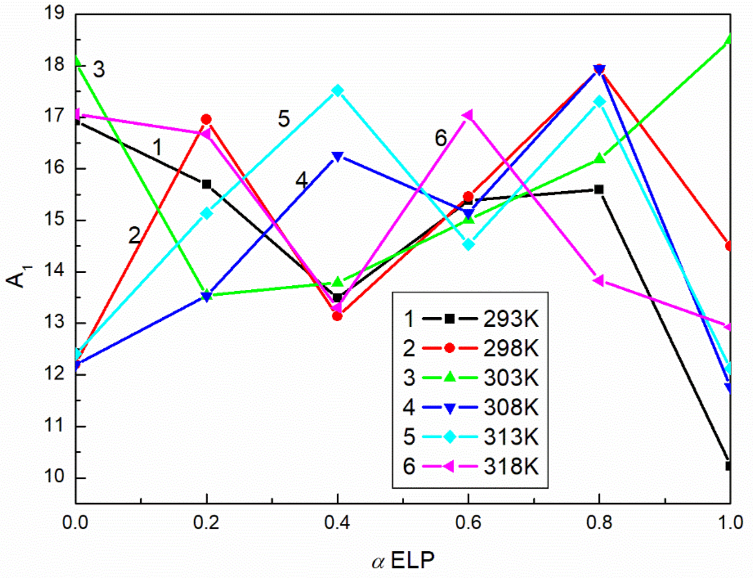

Figure S9. A plot of the values of constant A_1 in Eq. (1) for studied surfactant mixtures vs. the mole fraction of ELP in the bulk phase (α) at the temperatures equal to 293 (curve 1), 298 (curve 2), 303 (curve 3), 308 (curve 4), 313 (curve 5) and 318 K (curve 6), respectively.

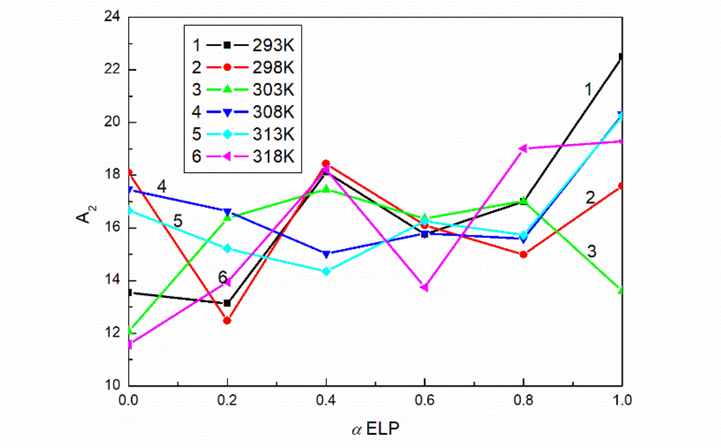

Figure S10. A plot of the values of constant A_2 in Eq. (1) for studied surfactant mixtures vs. the mole fraction of ELP in the bulk phase (α) at the temperatures equal to 293 (curve 1), 298 (curve 2), 303 (curve 3), 308 (curve 4), 313 (curve 5) and 318 K (curve 6), respectively.

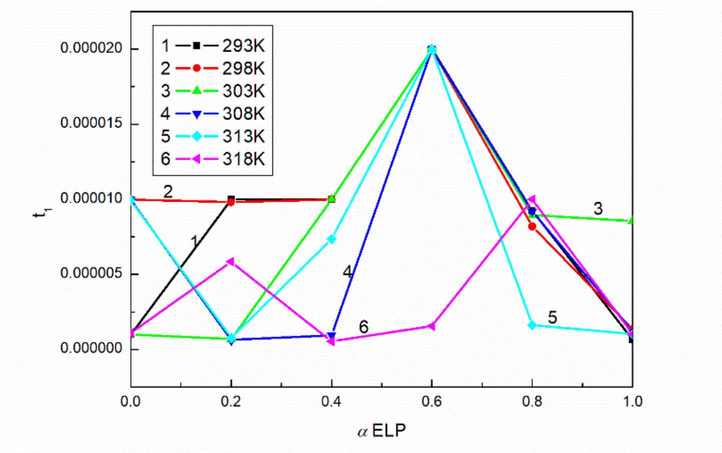

Figure S11. A plot of the values of constant t_1 in Eq. (1) for studied surfactant mixtures vs. the mole fraction of ELP in the bulk phase (α) at the temperatures equal to 293 (curve 1), 298 (curve 2), 303 (curve 3), 308 (curve 4), 313 (curve 5) and 318 K (curve 6), respectively.

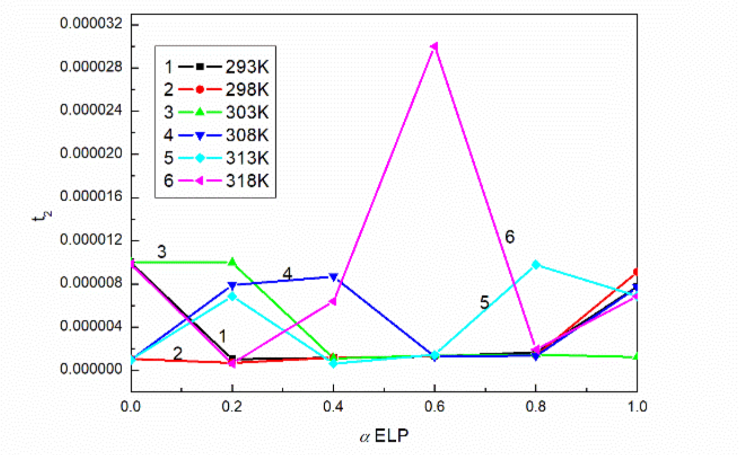

Figure S12. A plot of the values of constant t_2 in Eq. (1) for studied surfactant mixtures vs. the mole fraction of ELP in the bulk phase (α) at the temperatures equal to 293 (curve 1), 298 (curve 2), 303 (curve 3), 308 (curve 4), 313 (curve 5) and 318 K (curve 6), respectively.

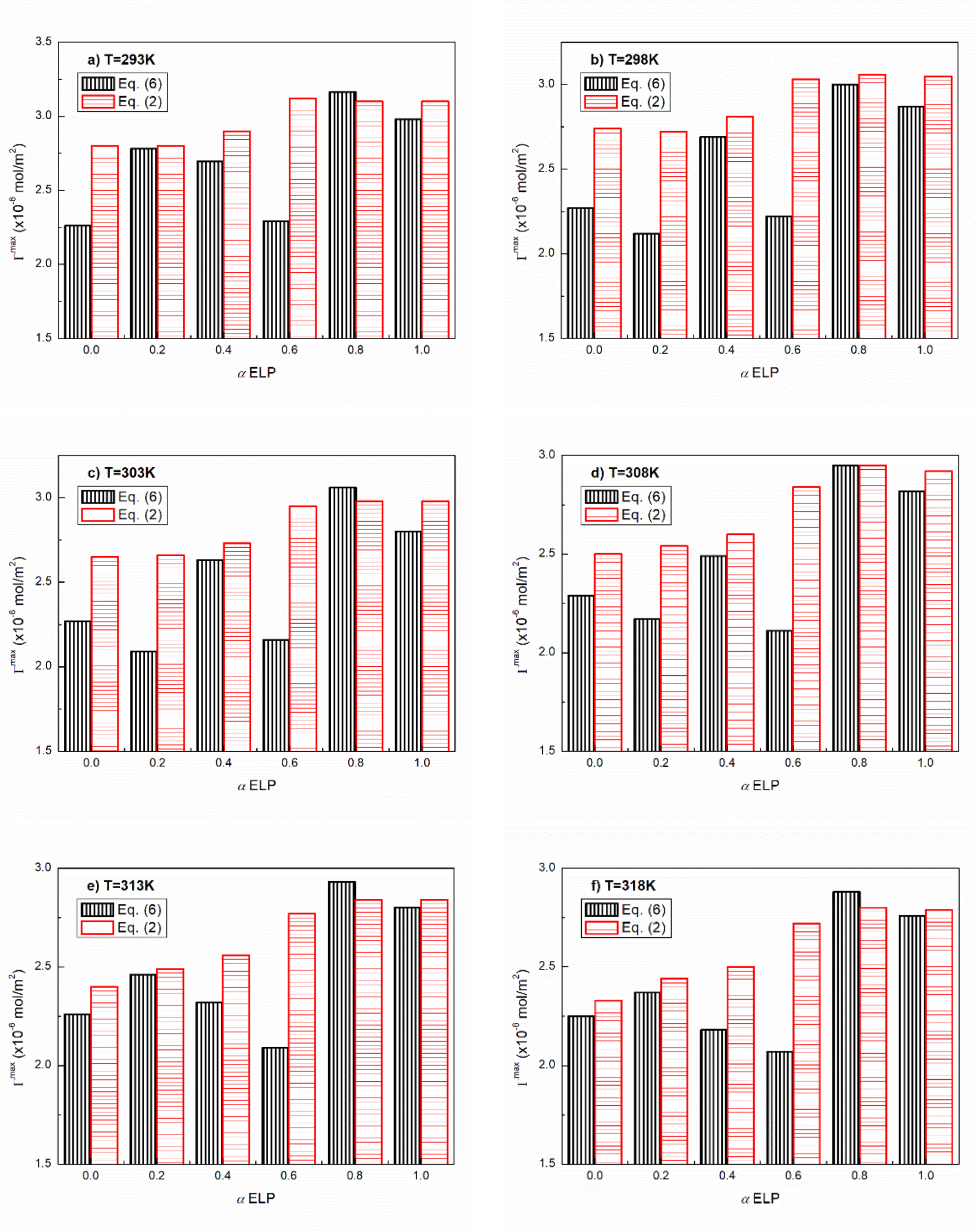

Figure S13. The values of Γ^{max} calculated from Eq. (6) and (2) for studied surfactant mixtures at the mole fraction of ELP in the bulk phase (α) equal to 0, 0.2, 0.4, 0.6, 0.8 and 1 at different temperatures equal to 293 (a), 298 (b), 303 (c), 308 (d), 313 (e) and 318 K (f), respectively.

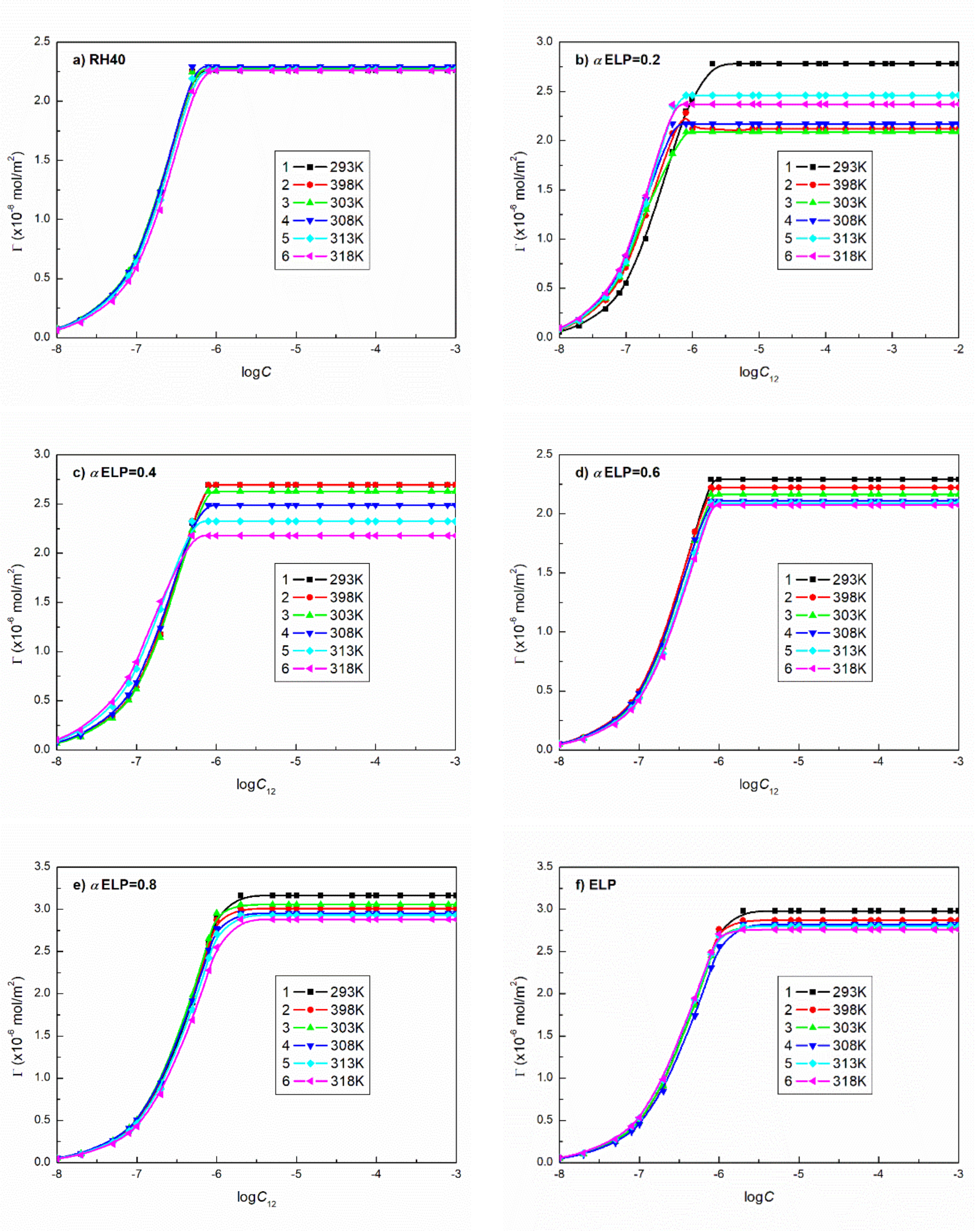

Figure S14. A plot of the values of Gibbs surface excess concentration (Γ) vs. logarithm of the concentration in the bulk phase (*C* or C_{12}) for the RH40 and ELP mixtures at the mole fraction of ELP in the bulk phase equal to 0 (RH40 (a)), 0.2 (b), 0.4 (c), 0.6 (d), 0.8 (e) and 1 (ELP (f)), respectively.

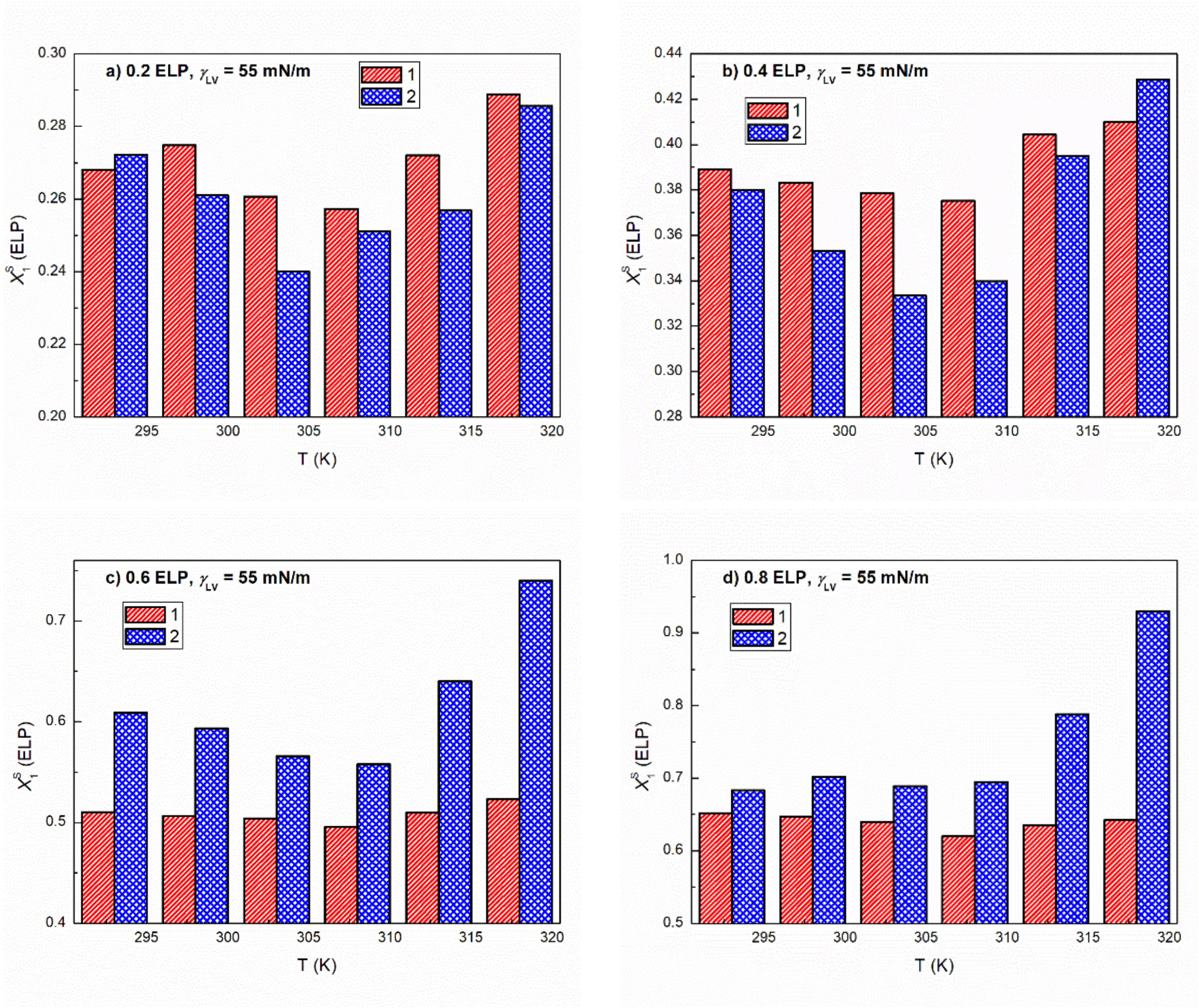

Figure S15. The values of the mole fraction of ELP in the mixed monolayer at the water air interface (X_1^S) calculated from the relationship: $X_1^S = \frac{\pi_1}{\pi_1+\pi_2}$ (bars 1) and from Eq. (8) (bars 2) and temperature range 293-318K for mixtures at the mole fraction of ELP in the bulk phase (α) equal to 0.2 (a), 0.4 (b), 0.6 (c) and 0.8 (d).

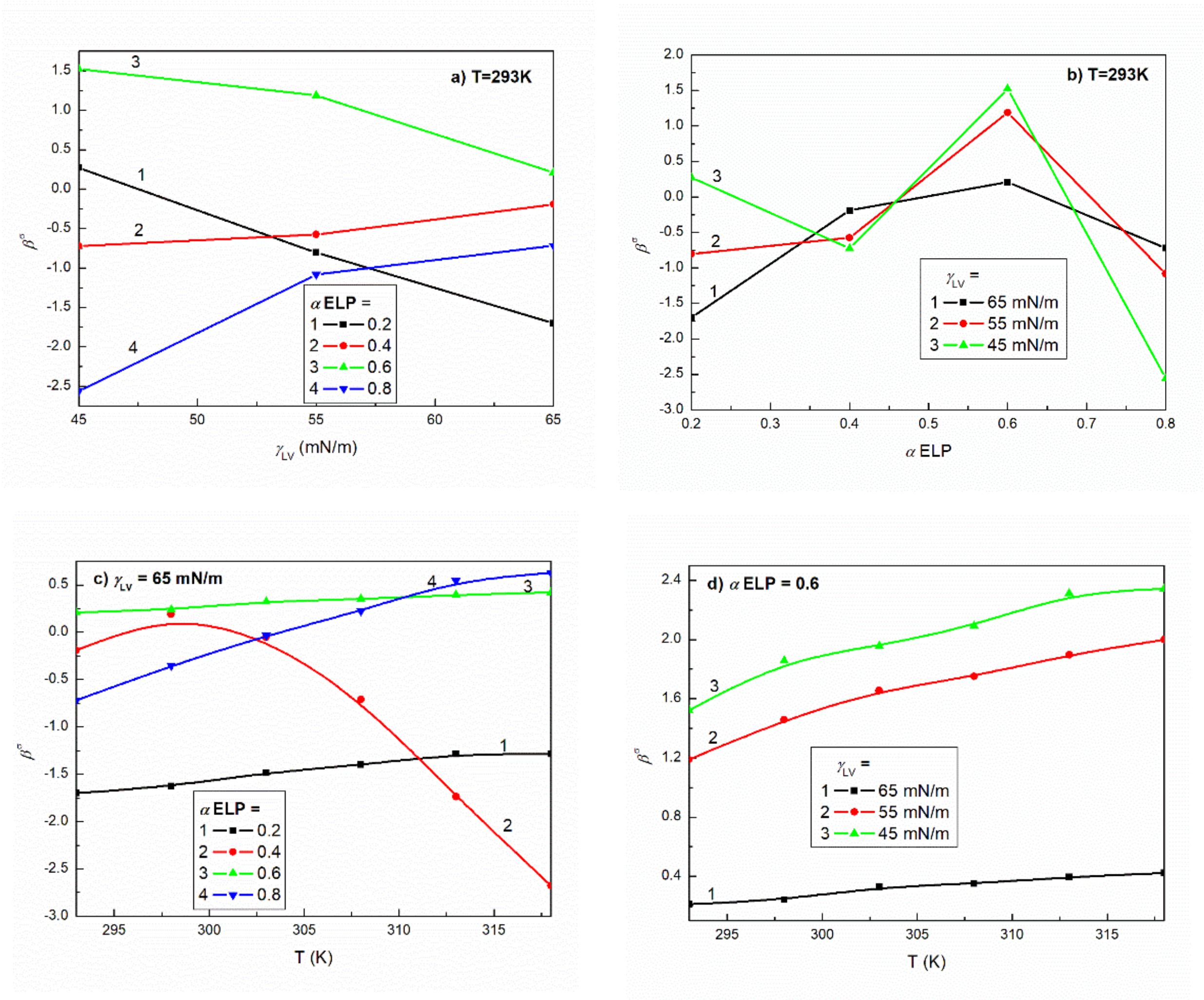

Figure S16. A plot of the values of parameter of intermolecular interactions, β^{σ}, for studied binary surfactant mixtures a) at the mole fraction of ELP in the bulk phase α, equal to 0.2, 0.4, 0.6, 0.8 and T = 293K vs. surface tension, (γ_{LV}), b) at the surface tension, (γ_{LV}), equal to 65, 55, 45 mN/m and T = 293K vs. mole fraction of ELP in the bulk phase α, c) at the mole fraction of ELP in the bulk phase α, equal to 0.2, 0.4, 0.6, 0.8 and γ_{LV} = 65 mN/m vs. temperature, T, d) at the mole fraction of ELP in the bulk phase α, equal to 0.6 and γ_{LV} = 65, 55, 45 mN/m vs. temperature, T.
